# Supplementary material for: The landscape of hepatocellular carcinoma recurrence after liver transplantation: A nationwide 14-year study
Source: JHEP Rep. 2026 Oct 9;8(12):102062. doi: 10.1016/j.jhepr.2026.102062 (PMC13770652; doi:10.1016/j.jhepr.2026.102062)
Supplement: Supplementary file 1 [file mmc1.pdf]

The landscape of hepatocellular carcinoma recurrence after liver transplantation: A nationwide 14-year study

Massih Ningarhari, Mathurine Leray, Audrey Coilly, Eric Vibert, Eric Assenat, Astrid Herrero, Jose Ursic-Bedoya, Camille Henry, Line-Carolle Ntandja-Wandji, Elise Lemaitre, Alexandre Louvet, Philippe Mathurin, Emmanuel Boleslawski, Fabien Robin, Thomas Uguen, Pauline Houssel-Debry, Camille Besch, François Faitot, Manon Allaire, Filomena Conti, Olivier Scatton, Giuliana Amaddeo, Daniele Sommacale, Jean-Baptiste Hiriart, Louis Marjary, Faiza Chermak, Laurence Chiche, Olivier Roux, Claire Francoz, Federica Dondero, Teresa Antonini, Domitille Erard, Jean-Marie Peron, Nassim Kamar, Bleuenn Brusset, Thomas Decaens, Laure Elkrief, Hélène Barraud, Ephrem Salamé, René Gerolami, Sophie Chopinet, Jérôme Dumortier, Olivier Boillot, Sébastien Dharancy, Guillaume Lassailly, on behalf of the GReF2 study group

Table of contents

Fig. S1 3
Fig. S2 4
Fig. S3. 5
Fig. S4 6
Fig. S5. 7
Table S1 8
Table S2 9
Table S3 10
Table S4 11
Table S5 12
Table S6 13
Table S7 14
Table S8. 15
Table S9 16
Table S10 17

Table S11 18

Table S12 19

Table S13 20

Fig. S1 – Study Flow-chart

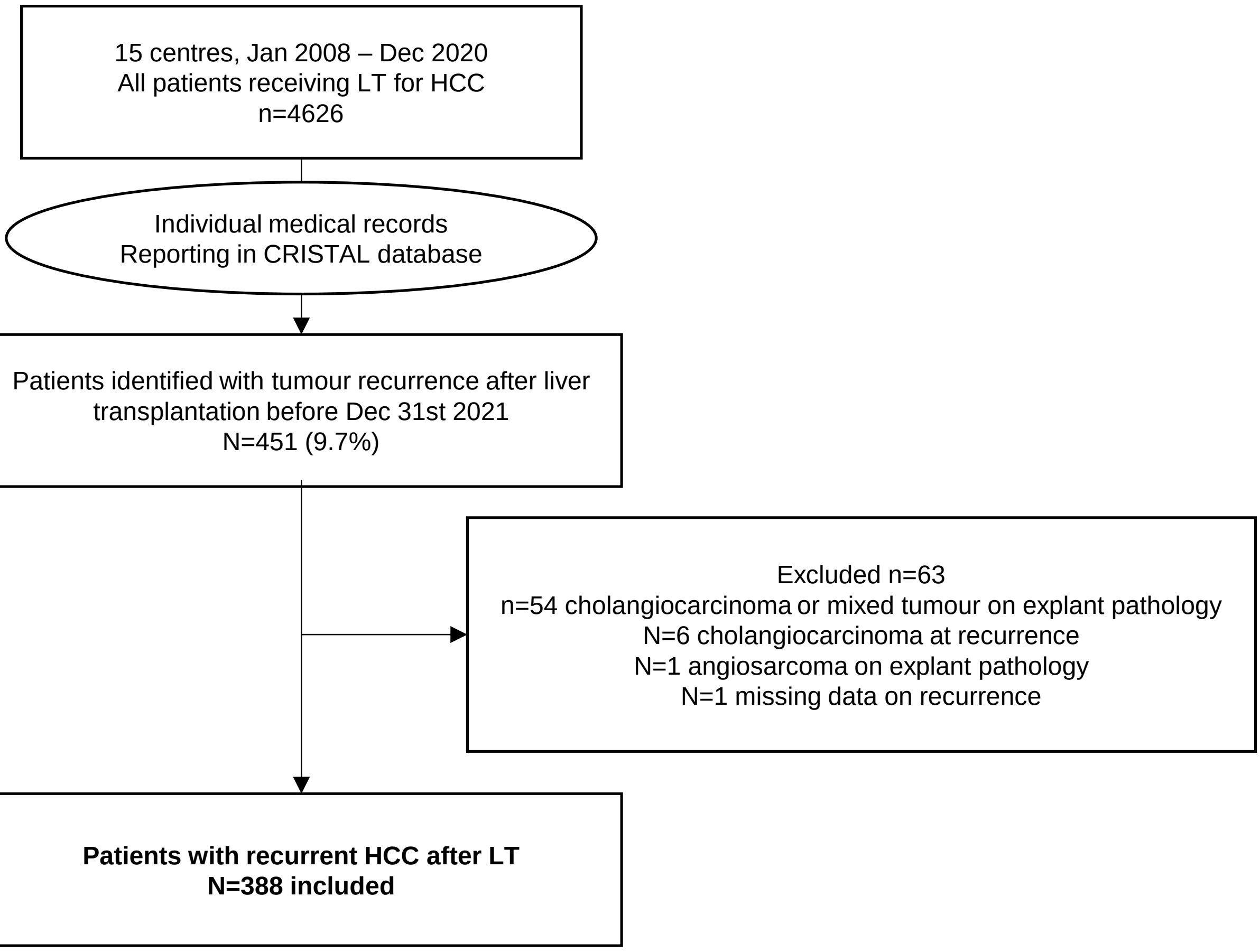

LT, liver transplantation. HCC, hepatocellular carcinoma.

Fig. S2 – Covariate balance after propensity score matching, to compare patients treated with curative versus non-curative intent

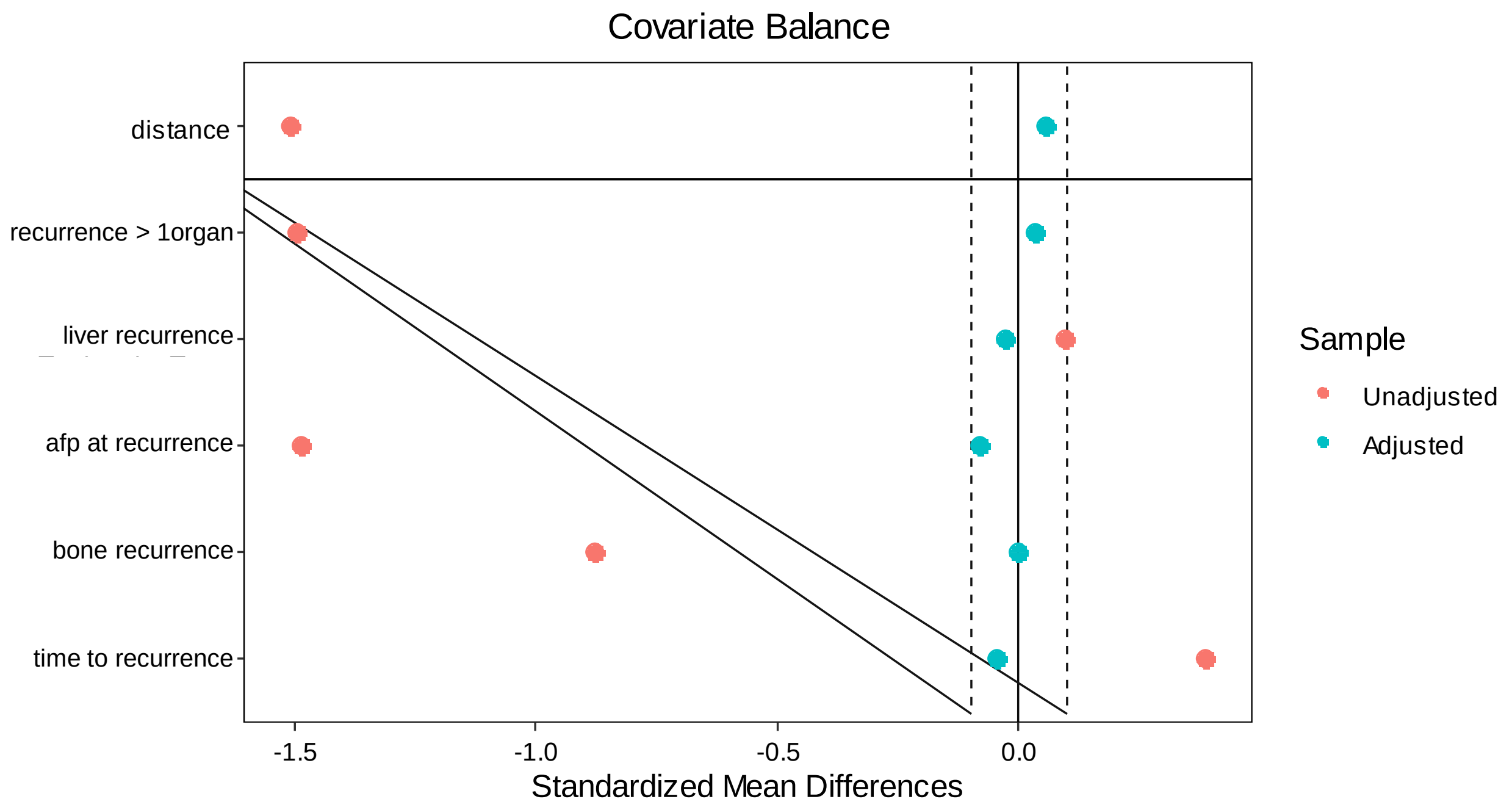

Fig. S3 – Benefit of treatments with curative intent in patients with only one organ affected by recurrence.

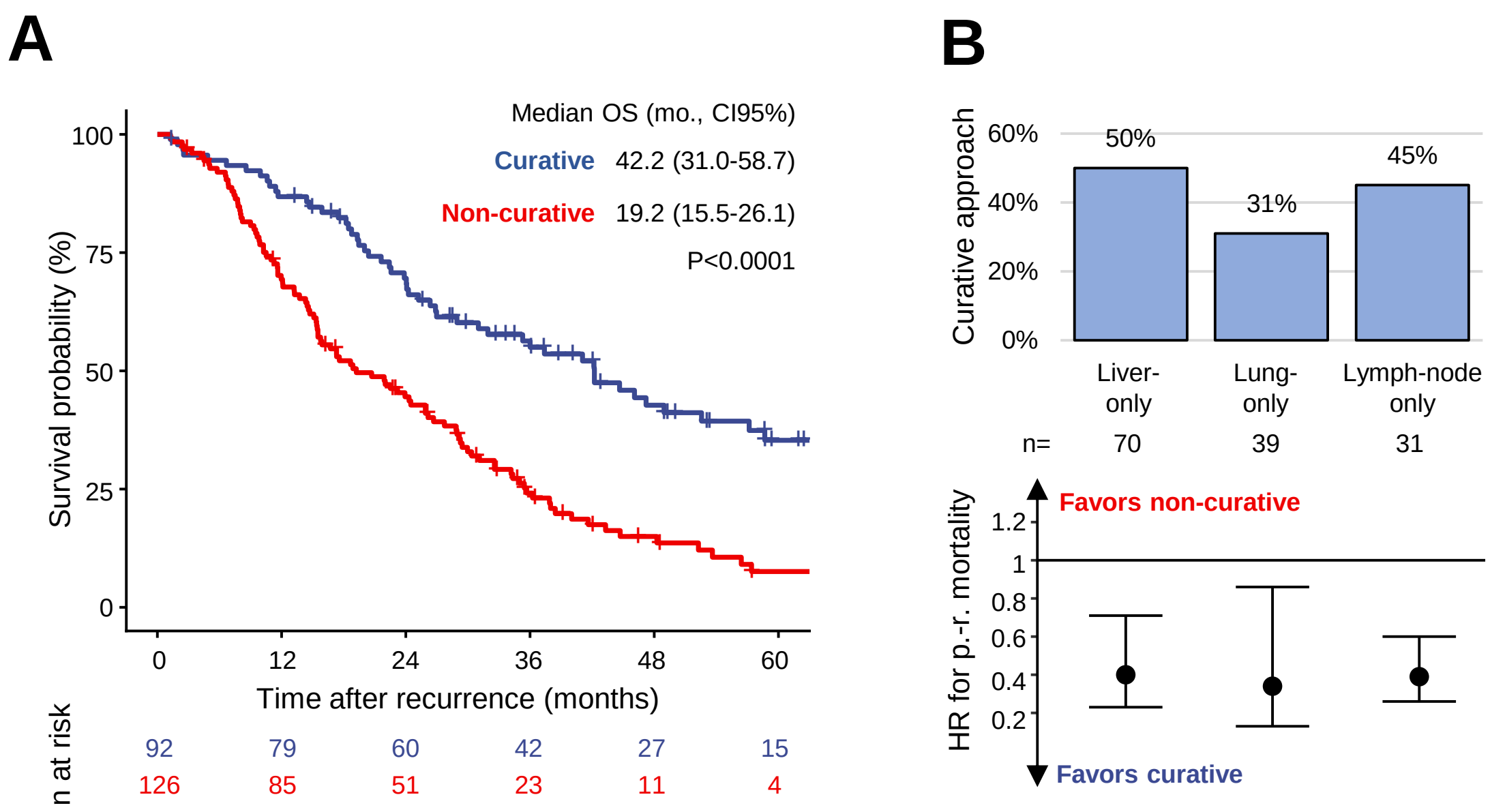

(A) Post-recurrence overall survival of patients according to the first treatment applied, compared with the log-rank test. (B) Proportion of patients (top) receiving surgical and ablative treatments, and benefit (bottom) of such treatment compared to systemic treatment first, according to the anatomical site of recurrence, using Cox regression model stratified by organ (dots represent the hazard ratio and bars the 95% confidence interval). Level of significance set at p≤0.05. n, number of patients with available data. OS, overall survival. Mo., months. CI95%, 95% confidence interval. NR, not reached. HR, hazard ratio. p-r., post-recurrence.

Fig. S4 – Distribution of scores according to the AFP model before and after the adoption as selection criteria in 2013

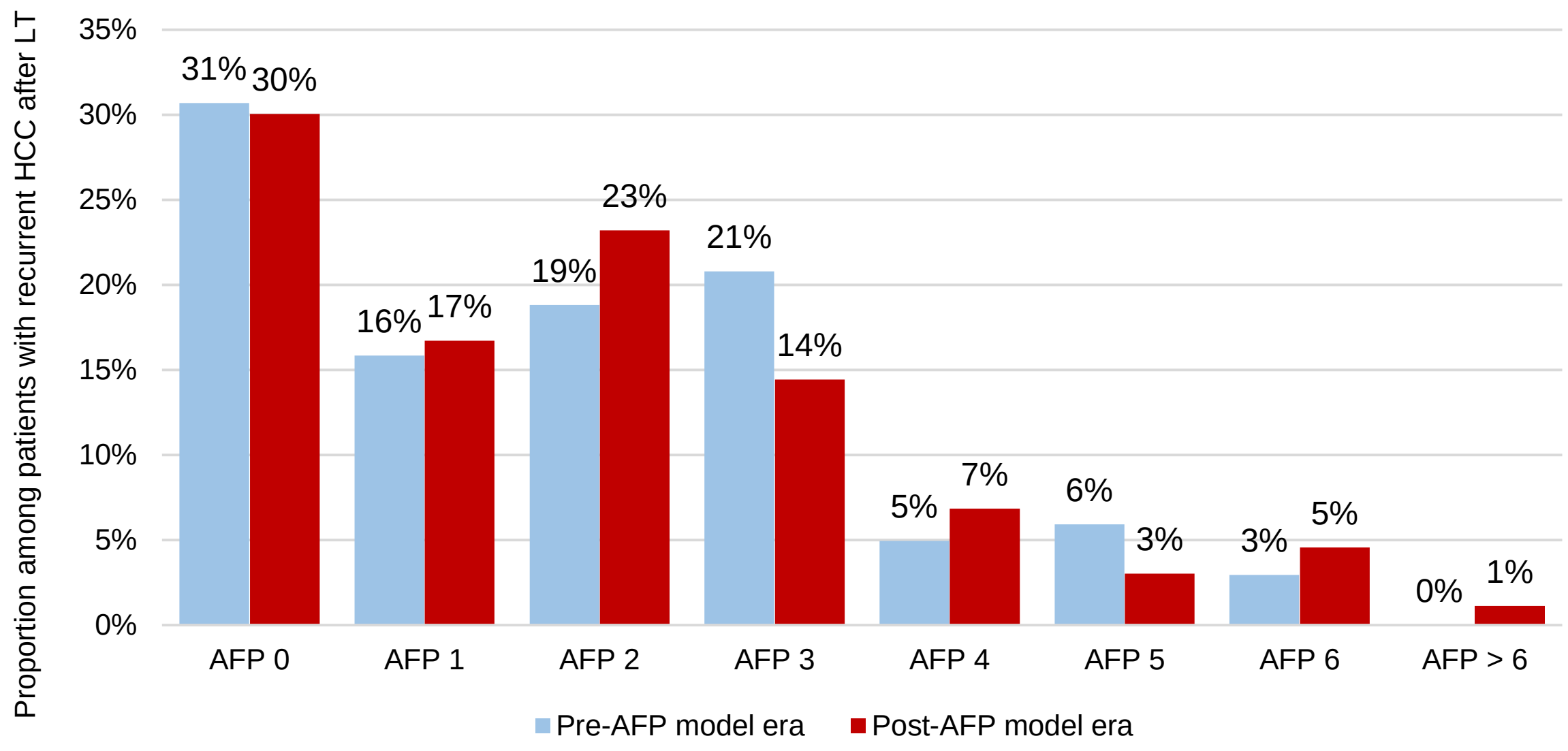

Fig. S5 – Association of pretransplant tumour characteristics with post-recurrence survival.

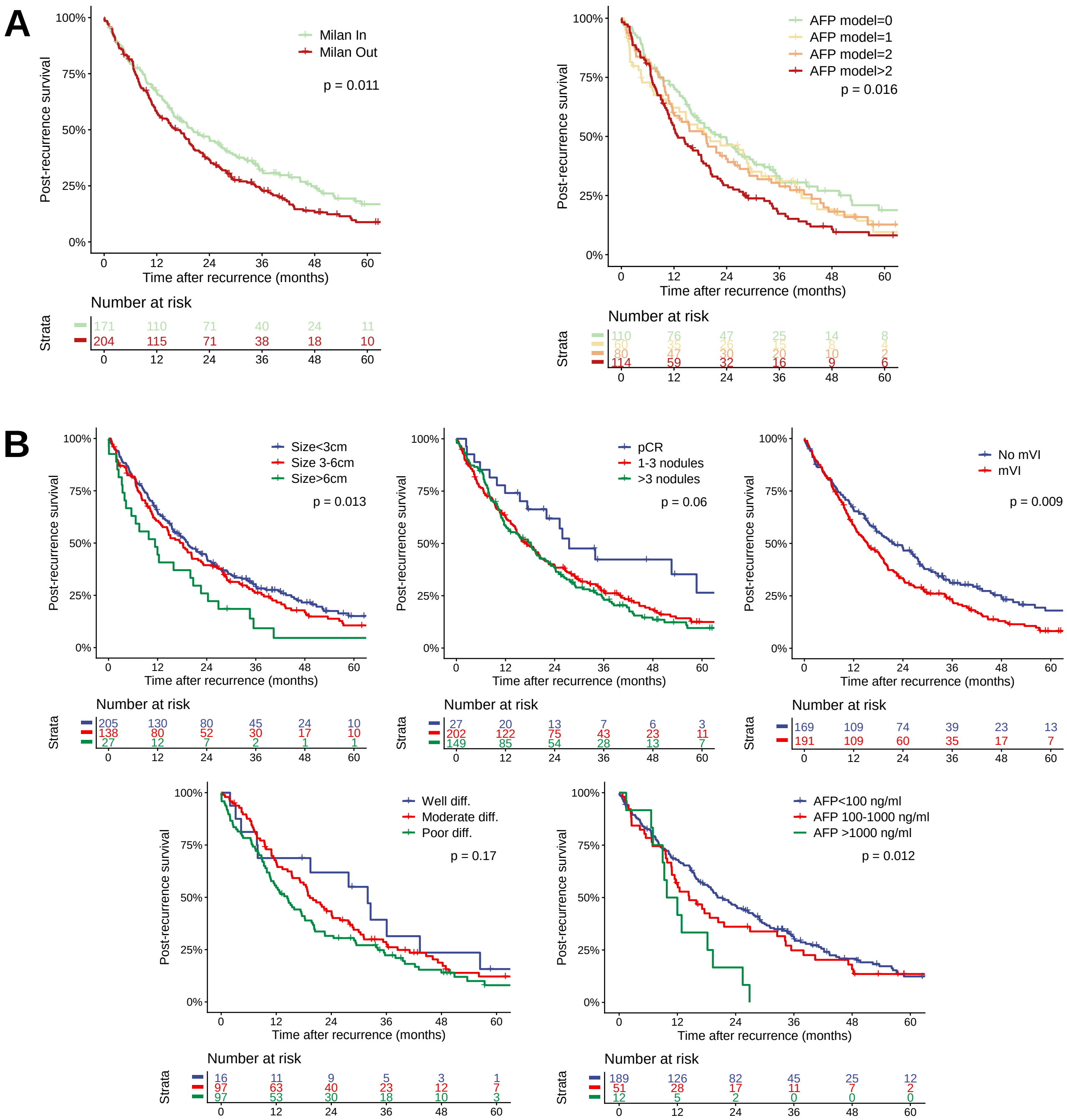

Post-recurrence overall survival according to (A) Milan criteria and AFP model score, and (B) individual components of the AFP model, vascular invasion and tumour differentiation, comparisons with the log-rank test. Level of significance set at p≤0.05. pCR, pathological complete response; mVI, microvascular invasion; diff., tumour differentiation

Table S1 – The AFP model criteria (adapted from Duvoux et al., Gastroenterology 2012)

Variables	β coefficient	Hazard ratio	Points
Largest diameter			
⩽3 cm	0	1	0
3–6 cm	0.272	1.31	1
>6 cm	1.347	3.84	4
Number of nodules			
1–3	0	1	0
4 and more	0.696	2.01	2
AFP level (ng/ml)			
⩽100	0	1	0
[100–1000]	0.668	1.95	2
>1000	0.945	2.57	3

The score is calculated by adding the individual points for each obtained variable. A cut-off of 2 separates between patients at high and low risk of recurrence.

Table S2 – Pre-transplant characteristics of patients with recurrent hepatocellular carcinoma after liver transplantation

	N	
**Male sex**	388	332 (85.6)
**Underlying liver disease and risk factors**		
Cirrhosis	388	367 (94.6)
Alcohol-related	388	209 (54.3)
BMI (kg/m²)	263	26.5 (23.3-30)
Diabetes	380	112 (29.5)
Hepatitis C	388	148 (38.1)
Hepatitis B	388	40 (10.3)
Other	388	21 (5.4)
**Time between HCC diagnosis to LT**	287	19.8 (12.5-33.1)
**Bridging therapy**		
Any	388	312 (80.4)
TACE	312	271 (86.9)
Other LRT (PA or EBRT)	312	69 (22.1)
Systemic	312	21 (6.7)
**Serum αFP dynamics on waitlist**		
αFP at listing (ng/ml)	359	8.9 (4.2-32)
Maximal αFP (ng/ml)	355	18 (6-85.5)
Last αFP pre-LT (ng/ml)	379	16 (5-100.5)
Stable αFP	355	171 (48.2)
Decreasing αFP >30%	355	66 (18.6)
Increasing αFP >20%	355	118 (33.2)
**Age at LT**	388	60.4 (55.2-63.8)
**LT in the AFP model era**	388	271 (70)

N, patients with available data. Quantitative variables are presented as median (interquartile range, IQR), qualitative variables as n (%). Percentage calculated within each line on patients with available data. BMI, body mass index. TACE, transarterial chemoembolization. LRT, locoregional therapy. PA, percutaneous ablation. EBRT, external-beam radiotherapy. αFP, alpha-foetoprotein. LT, liver transplantation.

Table S3 – Explant characteristics of patients with recurrent hepatocellular carcinoma after liver transplantation

	N	
**Number of nodules**	378	
0 (pathological complete response)		27 (7.1)
1-3		202 (53.4)
4+		149 (39.4)
**Size of the largest nodule**	370	
<3cm		205 (55.4)
3-6cm		138 (37.3)
>6cm		27 (7.3)
**Microvascular invasion**	360	191 (53.1)
**Nuclear grade 3**	210	97 (46.2)
**Milan-In**	375	171 (45.6)
**AFP model**	364	
0		110 (30.2)
1		60 (16.5)
2		80 (22.0)
>2		114 (31.3)

N, patients with available data. Qualitative variables are presented as n (%). Percentage calculated within each line on patients with available data.

Table S4 – Tumour burden at recurrence according to first treatment applied (all patients)

		Curative first (resection/ablation)	Locoregional first (incl. combination)	Systemic first	Best supportive care
	**N=**	**99**	**55**	**188**	**45**
**Organs**					
Liver	152	39 (25.7)	23 (15.1)	73 (48.0)	17 (11.2)
Lungs	123	15 (12.2)	6 (4.9)	82 (66.7)	20 (16)
Bones	100	9 (9.0)	16 (16.0)	55 (55.0)	20 (20.0)
Lymph nodes	92	15 (16.3)	8 (8.7)	58 (63.0)	11 (12.0)
Adrenal	29	11 (37.9)	2 (6.9)	14 (48.3)	2 (6.9)
Peritoneal/seeding	68	14 (20.6)	5 (7.4)	36 (52.9)	13 (19.1)
Others	55	16 (29.1)	10 (18.2)	23 (41.8)	6 (10.9)
**Distribution**					
Liver-only	77	35 (45.5)	14 (18.2)	22 (28.6)	6 (7.8)
Extrahepatic-only	235	60 (25.5)	32 (13.6)	115 (48.9)	28 (11.9)
Both	75	4 (5.3%)	9 (12.0)	51 (68.0)	11 (14.7)
**Number of organs**					
Single organ	238	92 (38.7)	42 (17.6)	84 (35.3)	20 (8.4)
Two organs	106	5 (4.7)	13 (12.3)	74 (69.8)	14 (13.2)
Three or more	42	2 (4.8)	0 (0)	29 (69.0)	11 (26.2)
**AFP at recurrence (ng/ml)**	340	8 (3-47)	36 (4-339)	14 (4-180)	29 (6-392)

N, patients with available data. Quantitative variables are presented as median (interquartile range, IQR), qualitative variables as n (%). BSC, best supportive care. Percentage calculated within each line on patients with available data.

Table S5 – Tumour burden at recurrence according to first treatment applied (patients with only one organ affected by recurrence, excluding those only eligible to best supportive care)

		**Curative first (resection/ablation)**	**Non-curative first (excl. BSC)**	
	**N**	**92**	**126**	**P**
**Organs**				
Liver	70	35 (50.0)	35 (50.0)	0.14
Lungs	39	12 (30.8)	27 (69.2)	0.16
Bones	36	5 (13.9)	31 (86.1)	0.0003
Lymph nodes	31	14 (45.2)	17 (54.8)	0.87
Adrenal	14	11 (78.6)	3 (21.4)	0.004
Peritoneal/seeding	15	10 (66.7)	5 (33.3)	0.09
Others	27	16 (59.3)	11 (40.7)	0.34
Extrahepatic-only	148	57 (38.5)	91 (61.5)	
**AFP at recurrence (ng/ml)**	196	8 (3-32)	11 (4-183)	0.13

N, patients with available data. Quantitative variables are presented as median (interquartile range, IQR), qualitative variables as n (%). BSC, best supportive care. Percentage calculated within each line on patients with available data. Comparisons and p values using $\chi 2$ test for qualitative variables, and Wilcoxon rank-sum (i.e. Mann-Whitney U-test) test for quantitative variables, level of significance set at $p \leq 0.05$.

Table S6 – Systemic therapies used for recurrent hepatocellular carcinoma after liver transplantation

	**L1**	**L2**	**L3**	**L4**	**L5**
**N=**	283	150	70	34	11
Sorafenib	232 (82.0)	14 (9.3)	1 (1.4)	0	2 (18.2)
Lenvatinib	28 (9.9)	9 (6.0)	5 (7.1)	10 (29.4)	5 (45.5)
Chemotherapy	14 (4.9)	36 (24.0)	25 (35.7)	6 (17.6)	2 (18.2)
Regorafenib	1 (0.4)	60 (40.0)	14 (20)	2 (5.9)	0
Cabozantinib	1 (0.4)	25 (16.7)	19 (27.1)	7 (20.6)	1 (9.1)
Immunotherapy	3 (1.1)	3 (2)	2 (2.9)	7 (20.6)	1 (9.1)
Others	4 (1.3)	3 (2)	4 (5.7)	2 (5.9)	0

N, patients with available data. Qualitative variables are presented as as n (%). Percentage calculated within each column on patients with available data. L1 to L5, First to fifth line of systemic treatment.

Table S7 - Characteristics of patients according to the year of recurrence of hepatocellular carcinoma after liver transplantation

Year of recurrence	2008-2015	2016-2017	2018-	p
N	133	109	146	
**Time to recurrence**				0.0007
<6 months	24 (18.0)	11 (10.1)	13 (8.9)	
6-36 months	82 (61.7)	70 (64.2)	86 (58.9)	
36-60 months	23 (17.3)	19 (17.4)	20 (13.7)	
>60 months	4 (3.0)	9 (8.3)	27 (18.4)	
**1st treatment for recurrence**				0.27
Curative intent	32 (24.0)	24 (22.0)	44 (30.1)	
Non-curative intent	101 (76.0)	85 (78.0)	102 (69.9)	
**Any systemic 1st-line**	**99 (74.4)**	**83 (76.1)**	**101 (69.2)**	0.35
Sorafenib	82 (82.8)	79 (95.2)	71 (70.3)	
Lenvatinib	2 (2.0)	2 (2.4)	24 (23.8)	
Other TKI	0	0	2 (2.0)	
IO	0	0	3 (3.0)	
Chemotherapy	13 (13.1)	2 (2.4)	1 (1.0)	
Other drugs	2 (2.0)	0	0	
**Access to 2nd line**	40 (40.4)	49 (59.0)	61 (60.4)	0.11
Sorafenib	9 (22.5)	0	5 (8.2)	
Lenvatinib	0	1 (2.0)	8 (13.1)	
Regorafenib	6 (15.0)	29 (59.2)	25 (41.0)	
Cabozantinib	1 (2.5)	6 (12.2)	18 (29.5)	
IO	0	0	3 (4.9)	
Chemotherapy	22 (55.0)	12 (24.5)	2 (3.3)	
Other drugs	2 (5.0)	1 (2.0)	0	
**Access to 3rd line**	17 (17.2)	26 (31.3)	27 (26.7)	0.27
**Access to >3 lines**	10 (10.1)	10 (12.0)	13 (12.9)	0.81

N, number of patients with available data. Qualitative variables are presented as n (%). Percentage are calculated within each column on patients with available data. HCC, hepatocellular carcinoma. TKI, tyrosine kinase inhibitor. IO, immunotherapy (single or combination). Comparisons and p values using $\chi2$ test for qualitative variables, level of significance set at $p \leq 0.05$.

Table S8 – Main characteristics of patients diagnosed after 2018 and treated with either sorafenib or lenvatinib as first-line systemic therapy.

	**N**	**Sorafenib 71**	**Lenvatinib 24**	**P**
**Time to recurrence (months)**	95	23.8 (13.4-36.8)	29.5 (11.1-49.9)	0.68
**Delay of recurrence**	95			0.4
<6 months		3 (4.2)	2 (8.3)	
6-36 months		49 (69)	13 (54.2)	
36-60 months		10 (14.1)	3 (12.5)	
>60 months		9 (12.7)	6 (25)	
**Time from recurrence to systemic therapy initiation**	95	2 (0.9-3.8)	6.5 (2.1-17.5)	0.008
**Recurrent liver disease**	93	10 (14.5)	3 (12.5)	
**Recurrent tumour distribution**	95			
Liver		29 (40.8)	3 (12.5)	0.02
Lungs		17 (23.9)	7 (29.2)	0.84
Bones		21 (29.6)	8 (33.3)	0.96
>1 organ affected		38 (53.5)	5 (20.8)	0.009
Liver-only		12 (16.9)	2 (8.3)	
Extrahepatic-only		42 (59.2)	21 (87.5)	
Both		17 (23.9)	1 (4.2)	
**AFP at recurrence (ng/ml)**	90	14 (5-94)	7 (3-64)	0.15
**Previous treatment**	95			
Surgery/Ablation		7 (9.9)	9 (37.5)	0.005
Non-curative locoregional treatment		9 (12.7)	7 (29.2)	0.12

N, patients with available data. Quantitative variables are presented as median (interquartile range, IQR), qualitative variables as n (%). Percentage calculated within each column on patients with available data. Comparisons and p values using $\chi 2$ test for qualitative variables, and Wilcoxon rank-sum (i.e. Mann-Whitney U-test) test for quantitative variables, level of significance set at $p \leq 0.05$.

Table S9 – Sensitivity analysis on patients with patients receiving LT before 2016, restricted on a 5-year post-LT window

**Selection era for LT**	**Before AFP model**	**After AFP model**	
**N**	**90**	**154**	**p**
**Time to recurrence**			
<6 months	13 (14.4)	17 (11.0)	**0.02**
6-36 months	48 (53.3)	109 (70.8)	
36-60 months	29 (32.2)	28 (18.2)	

N, patients with available data. Qualitative variables are presented as n (%). Percentage on patients with available data within each era of transplantation. LT, liver transplantation. Comparisons and p values using $\chi 2$ test for qualitative variables, level of significance set at $p \leq 0.05$.

Table S10 – Patients characteristics according to mTOR inhibitor introduction before recurrence

		mTORi before recurrence	No mTORi before recurrence	
	N=	104	266	P
**Tumour dynamics before LT**				
Any bridging therapy	370	86 (82.7)	211 (79.3)	0.56
AFP dynamics				
AFP at listing	344	9 (4-38)	9 (4-32)	0.97
Maximal AFP	339	23 (7-168)	16 (5-69)	0.03
Last AFP pre-LT	362	14 (6-88)	10 (4-46)	0.02
Stable AFP		39 (41.1)	124 (50.8)	0.24
Decreasing AFP >30%		22 (23.2)	43 (17.6)	
Increasing AFP >20%		34 (35.8)	77 (31.6)	
**Explant pathology**				
Number of nodules	361			<0.0001
0 (pCR)		2 (2)	25 (9.7)	
1-3		39 (38.2)	153 (59.1)	
4+		61 (59.8)	81 (31.3)	
Size of the largest nodule	353			0.1
<3cm		48 (48.5)	150 (59.1)	
3-6cm		40 (40.4)	89 (35)	
>6cm		11 (11.1)	15 (5.9)	
Microvascular invasion	343	59 (60.2)	121 (49.4)	0.09
Nuclear grade 3	199	29 (40.3)	61 (48)	0.47
Milan-In using explant	358	26 (25.5)	140 (54.7)	<0.0001
AFP model using explant	348			<0.0001
0		15 (15.5)	92 (36.7)	
1		11 (11.3)	46 (18.3)	
2		28 (28.9)	49 (19.5)	
>2		43 (44.3)	64 (25.5)	
**LT characteristics**				
Age at LT	370	60 (53-64)	61 (56-64)	0.54
LT after AFP model implementation	370	80	183	0.16
**IS management before recurrence**				
Time from LT to mTORi (months)		2.6 (1.6-7)	-	-
Time from mTORi to recurrence (months)		14.9 (6-26.1)	-	-

N, patients with available data. Quantitative variables are presented as median (interquartile range, IQR), qualitative variables as n (%). Percentage on patients with available data within each column. Comparisons and p values using $\chi 2$ test for qualitative variables, and Wilcoxon rank-sum (i.e. Mann-Whitney U-test) test for quantitative variables, level of significance set at $p \leq 0.05$. LT, liver transplantation. mTORi, mTOR inhibitor. pCR, pathological complete response.

Table S11 – Combined mTOR and calcineurin inhibitors management

		CNI interrupted before recurrence	CNI interrupted upon recurrence	CNI maintained after recurrence
	**N=293**	**36**	**71**	**186**
mTORi before recurrence	**70**	30 (42.9)	9 (12.9)	31 (44.3)
mTORi upon recurrence	**126**	4 (3.2)	61 (48.4)	61 (48.4)
mTORi not introduced	**97**	2 (2.1)	1 (1)	94 (96.9)

N, patients with available data. Qualitative variables are presented as n (%). Percentage on patients with available data within each line. mTORi, mTOR inhibitor. CNI, calcineurin inhibitor.

Table S12 – Characteristics at recurrence according to the use of mTOR inhibitors in post-recurrence immunosuppressive regimen

		**With mTOR inhibitor**	**Without mTOR inhibitor**	
	**N=**	**257**	**113**	**P**
**Time to recurrence (months)**	370	18 (9.8-34.3)	20.5 (11.8-46)	0.05
**Delay of recurrence**	370			0.12
<6 months		31 (12.1)	14 (12.4)	
6-36 months		169 (65.8)	62 (54.9)	
36-60 months		36 (14)	20 (17.7)	
>60 months		21 (8.2)	17 (15)	
**Age at recurrence**	370	62 (56-66)	63 (59-67)	0.04
**Recurrent liver disease**	357	43 (17.6)	20 (17.9)	1
**Recurrent tumour distribution**	370			
Liver		94 (36.6)	51 (45.1)	0.15
Lungs		79 (30.7)	39 (34.5)	0.57
Bones		66 (25.7)	29 (25.7)	1
>1 organ affected		91 (35.4)	51 (45.1)	0.1
Liver-only		52 (20.2)	22 (19.5)	0.1
Extrahepatic-only		163 (63.4)	62 (54.9)	
Both		42 (16.3)	29 (25.7)	
**AFP at recurrence**	327	14 (4-130)	9 (3-208)	0.65
**Treatment with curative intent**	370	66 (25.7)	28 (24.8)	0.96

N, patients with available data. Quantitative variables are presented as median (interquartile range, IQR), qualitative variables as n (%). Percentage on patients with available data within each column. Comparisons and p values using $\chi 2$ test for qualitative variables, and Wilcoxon rank-sum (i.e. Mann-Whitney U-test) test for quantitative variables, level of significance set at $p \leq 0.05$. LT, liver transplantation.

Table S13 – Factors associated with mortality after recurrence of hepatocellular carcinoma after liver transplantation (Cox regression)

	N	% missing	Univariable HR (CI95%)	Univariable p	Multivariable HR (CI95%)	Multivariable p
Age	388	0%	1 (0.98-1.01)	0.9	-	-
LT after AFP Criteria implementation	388	0%	1.13 (0.89-1.44)	0.3	-	-
Date of recurrence	388	0%				
2008-2015			Ref.		-	-
2015-2017			1.1 (0.84-1.43)	0.5	-	-
2018-			0.8 (0.61-1.05)	0.1	-	-
**Time to recurrence**	**388**	**0%**				
**<6 months**			**Ref.**		**Ref.**	
**6-36 months**			**0.63 (0.45-0.89)**	**0.007**	**0.59 (0.41-0.84)**	**0.004**
**36-60 months**			**0.4 (0.26-0.62)**	**<0.001**	**0.44 (0.27-0.69)**	**<0.001**
**>60 months**			**0.36 (0.21-0.61)**	**<0.001**	**0.34 (0.19-0.60)**	**<0.001**
PS grade >0	347	11%	2.27 (1.79-2.89)	<0.001	-	-
Serum αFP (by $\log^{10}$)	340	12%	1.37 (1.23-1.54)	<0.001	-	-
Organs affected by recurrence						
**Liver**	**388**	**0%**	**1.38 (1.1-1.73)**	**0.006**	**1.56 (1.19-2.04)**	**0.001**
Lungs	388	0%	1.24 (0.98-1.57)	0.07	-	-
**Bones**	**388**	**0%**	**1.71 (1.33-2.19)**	**<0.001**	**1.50 (1.14-1.98)**	**0.004**
**More than one organ**	**388**	**0%**	**2.02 (1.6-2.54)**	**<0.001**	**1.36 (1.04-1.78)**	**0.03**
Distribution of recurrence	388	0%				
Liver-only			Ref.		-	-
Extrahepatic only			0.86 (0.65-1.15)	0.32	-	-
Both			1.46 (1.02-2.07)	0.04	-	-
**Treatment with curative intent**	**387**	**0%**	**0.33 (0.24-0.44)**	**<0.001**	**0.43 (0.31-0.6)**	**<0.001**
**mTOR inhibitor**	**370**	**5%**	**0.67 (0.52-0.86)**	**0.001**	**0.62 (0.48-0.79)**	**<0.001**
CNI	295	24%	1.25 (0.96-1.63)	0.1	-	-
MMF	293	24%	1.12 (0.86-1.45)	0.4	-	-

The most relevant variables with rates of missing data <10% and significant association (threshold set p<0.05) with mortality in univariate analysis were introduced into the multivariate Cox model, level of significance set at p≤0.05. N, patients with available data. HR, hazard ratio. CI95%, 95% confidence interval. PS, performance status. CNI, calcineurin inhibitor. MMF, mycophenolate mofetil.
