# Supplementary material for: The landscape of hepatocellular carcinoma recurrence after liver transplantation: A nationwide 14-year study
Source: JHEP Rep. 2026 Oct 9;8(12):102062. doi: 10.1016/j.jhepr.2026.102062 (PMC13770652; doi:10.1016/j.jhepr.2026.102062)
Supplement: Supplementary file 3 [file mmc4.pdf]

JHEP Reports

The landscape of hepatocellular carcinoma recurrence after liver transplantation: A nationwide 14-year study

Authors

Massih Ningarhari, Mathurine Leray, Audrey Coilly, …, Olivier Boillot, Sébastien Dharancy, Guillaume Lassailly

Correspondence

massih.ningarhari@chu-lille.fr (M. Ningarhari), guillaume.lassailly@chu-lille.fr (G. Lassailly).

Graphical abstract

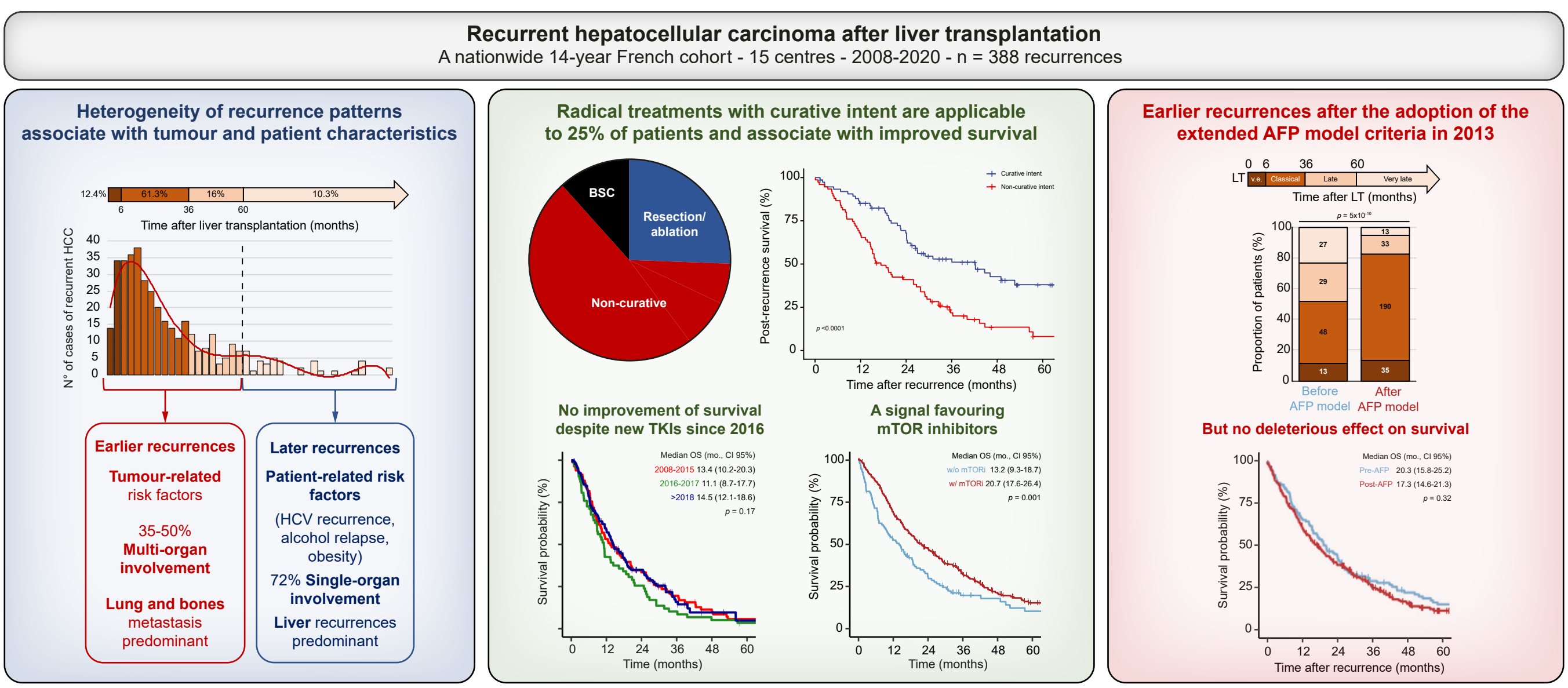

Highlights:

- Recurrent HCC after LT is highly heterogeneous in terms of timing and anatomical distribution.
- Recurrences beyond 3 years after LT occurred in about 30% of cases and were associated with patient-related risk factors.
- Implementation of extended criteria (AFP model) was associated with earlier recurrences.
- Curative treatments lead to better post-recurrence survival in selected patients with single organ involvement.
- Approval of second-generation TKIs after 2016 did not incrementally improve survival.

Impact and implications:

This nationwide cohort study provides contemporary data on hepatocellular carcinoma (HCC) recurrence after liver transplantation, a population excluded from major HCC trials. Recurrence patterns and outcomes were heterogeneous, and survival did not improve significantly across treatment eras despite the introduction of second-generation tyrosine kinase inhibitors after 2016. These findings may help transplant hepatologists and liver oncologists refine patient selection, individualise surveillance beyond the 3-year window recommended by the ILTS-ILCA based on risk factors for late recurrence, and prioritise curative-intent treatments in selected patients with disease limited to a single site. The retrospective design precludes definitive conclusions about the superiority of therapeutic strategies, but the findings can inform clinical trials evaluating newer systemic agents, including immune checkpoint inhibitors.

https://doi.org/10.1016/j.jhepr.2026.102062

© 2026 The Authors. Published by Elsevier B.V. on behalf of European Association for the Study of the Liver (EASL). This is an open access article under the CC BY license (http://creativecommons.org/licenses/by/4.0/).

The landscape of hepatocellular carcinoma recurrence after liver transplantation: A nationwide 14-year study

Massih Ningarhari[1,2,*,†], **Mathurine Leray**[1,†], Audrey Coilly[3], Eric Vibert[3], Eric Assenat[4], Astrid Herrero[5], Jose Ursic-Bedoya[6], Camille Henry[1], Line-Carolle Ntandja-Wandji[1,2], Elise Lemaitre[1], Alexandre Louvet[1,2], Philippe Mathurin[1,2], Emmanuel Boleslawski[7], Fabien Robin[8], Thomas Uguen[9], Pauline Houssel-Debry[9], Camille Besch[10], François Faitot[11], Manon Allaire[12], Filomena Conti[13], Olivier Scatton[14], Giuliana Amaddeo[15], Daniele Sommacale[16], Jean-Baptiste Hiriart[17], Louis Marjary[17], Faiza Chermak[17], Laurence Chiche[18], Olivier Roux[19], Claire Francoz[19], Federica Dondero[20], Teresa Antonini[21], Domitille Erard[21], Jean-Marie Peron[22], Nassim Kamar[23], Bleuenn Brusset[24], Thomas Decaens[24], Laure Elkrief[25], Hélène Barraud[25], Ephrem Salamé[26], René Gerolami[27], Sophie Chopinet[28], Jérôme Dumortier[29], Olivier Boillot[29], Sébastien Dharancy[1,2], Guillaume Lassailly[1,*], on behalf of the GReF2 study group

JHEP Reports **2026**. vol. 8 | 1–14

Check for updates

Background & Aims: Recurrence of hepatocellular carcinoma (r-HCC) remains a major determinant of post-transplant (LT) outcomes. The 2024 ILTS-ILCA consensus highlighted the need for contemporary data assessing how evolving selection criteria and therapeutic strategies over the past 15 years have influenced prognosis.

Methods: We conducted a retrospective study including all patients diagnosed with r-HCC following LT between 2008 and 2020 across 15 French transplant centres. Recurrence patterns, post-recurrence survival, and overall transplant outcomes were analysed, including temporal trends and the impact of AFP model implementation in 2013.

Results: We analysed 388 patients with r-HCC. The median time to recurrence was 19.1 months, with 26.3% of recurrences occurring beyond 3 years. Earlier recurrence was associated with tumour-related factors (AFP score, Milan Criteria, AFP kinetics), whereas later recurrence was associated with patient-related factors (HCV recurrence, alcohol relapse, and grade 2 obesity). Following implementation of the AFP model, time to recurrence decreased (p <0.001). Surgical or ablative treatments were associated with improved survival in a subgroup of patients with disease involving a single organ (liver, lung, or lymph nodes). Survival did not differ significantly among patients diagnosed before 2016, between 2016 and 2018, or after 2018 (p = 0.09). Calcineurin inhibitors and mycophenolate mofetil had no impact on survival, whereas mTOR inhibitor use was associated with significantly longer survival (20.7 *vs.* 13.2 months, p = 0.001).

Conclusions: r-HCC is a heterogeneous disease distinct from HCC in non-transplanted patients. The reduced median time to recurrence following implementation of expanded selection criteria warrants caution. Surgical or ablative approaches should be prioritised, including for extrahepatic disease. Despite the expansion of TKI treatment options since 2016, outcomes following systemic treatment did not improve significantly. The role of post-recurrence mTOR inhibitors should be evaluated in clinical trials.

© 2026 The Authors. Published by Elsevier B.V. on behalf of European Association for the Study of the Liver (EASL). This is an open access article under the CC BY license (http://creativecommons.org/licenses/by/4.0/).

Introduction

Hepatocellular carcinoma (HCC) is the second leading indication for liver transplantation (LT) in Europe, accounting for approximately 20% of the 7,000 LTs performed each year.[1] The individual benefit of LT is achieved through the combined treatment of both the solid tumour and the underlying liver disease. In the context of organ shortage, the allocation of this scarce resource is prioritized to patients with the best expected outcomes, based on overall post-transplantation survival as the main endpoint. To date, HCC recurrence has been considered as a reliable surrogate endpoint, because of its dismal prognosis. Therefore, selection criteria for LT in patients with HCC have been developed mainly to predict the risk of HCC recurrence as a binary outcome.

Since the first Milan criteria in 1996, major shifts, both in patient selection for LT and in treatment options for HCC, have led to new uncertainties regarding the natural history and prognosis of HCC recurrence after LT. The use of “extended criteria”, as acknowledged by the updated 2022 BCLC classification[2] and 2024 EASL guidelines,[3] allows for LT in a subset of patients exceeding the traditional Milan criteria. In France, the “AFP model”, which incorporates serum alpha-fetoprotein (AFP) concentration in addition to tumour burden, has replaced

* Corresponding authors. Address: CHU Lille, Hôpital Huriez, Maladies de l’Appareil Digestif, 2 rue Michel Polonovski, 59037 Lille, France. Tel.: +33 3 20 44 53 21; fax: +33 3 20 44 53 43.
E-mail addresses: massih.ningarhari@chu-lille.fr (M. Ningarhari), guillaume.lassailly@chu-lille.fr (G. Lassailly).
† Share co-first authorship.
https://doi.org/10.1016/j.jhepr.2026.102062

the Milan criteria as the recommended selection model since 1 March 2013.[4] Under this model, exception points are not awarded when the AFP score is >2. This led to the extension of tumour burden criteria beyond Milan criteria, as nodule size could be up to 6 cm, and the number of nodules was not a factor that could solely contraindicate LT anymore. The risk of HCC recurrence did not increase following the change in allocation policy.[5] However, whether the implementation of such extended criteria can impact the characteristics and prognosis of HCC recurrence is currently unknown.

The ILTS-ILCA guidelines regarding surveillance after LT consider that early detection of HCC recurrence "likely" improves survival.[6] Therefore, experts currently recommend a "reasonable" strategy of biannual chest and abdominal imaging. However, they also acknowledge the scarcity of data to support such a recommendation, and that the benefit of aggressive local therapies, for a disease that is metastatic *per se* when recurring after LT, remains to be clarified.

The systemic treatment landscape of HCC has seen major improvements in the past 15 years, including the approval of sorafenib in 2008, the development of several tyrosine kinase inhibitors (TKIs) after 2016, and immune checkpoint inhibitor-based combinations as first-line options since 2020. However, transplanted patients have always been excluded from pivotal trials, generating uncertainty regarding the efficacy and safety of those treatment options in this special population. In the wake of emerging questions regarding the risks and benefits of introducing ICI-based combinations in transplanted patients, contemporary data on recurrent HCC is needed. Whether the improvements in overall survival (OS) obtained in non-transplanted patients can also be replicated in transplanted patients with HCC is still unclear.

The use of immunosuppressive agents to prevent allograft rejection adds another layer of complexity, as they are also suspected to promote tumour progression. There is currently insufficient evidence to support any recommendation for a specific immunosuppressive regimen in patients with recurrent HCC after LT.

Given these uncertainties, recent ILTS-ILCA guidelines advocate for additional data in this special population. In the present study, we therefore aimed to provide such updated data in a large cohort of patients who have developed HCC recurrence after LT performed from 2008 to 2020, in a homogenous allocation framework of a single country, analysing the impacts of a national and coordinated shift to extended selection criteria in 2013 and of gradual improvements in HCC treatments.

Patients and methods

Study conduct

The study was conducted retrospectively in 15 out of the 17 tertiary centres performing LT in France: Lille, Bordeaux, APHP Paul-Brousse, Beaujon, Mondor, and Pitié-Salpêtrière, Tours, Montpellier, Strasbourg, Toulouse, Rennes, Marseille, Grenoble, Lyon Croix-Rousse and Edouard-Herriot. All variables collected were derived from routine care and recorded in digital medical records in each centre. The data were merged in a pseudonymised online database ('RedCap'), stored on a server under the responsibility of the Lille University Hospital, and protected from access by nominative identifiers. The collection and analysis of the data were authorized by the CNIL, the French data protection authority, through the local data protection authority of Lille University Hospital (authorization n°DEC22-074), in accordance with French regulations on retrospective non-interventional research involving existing data.

Patients

Patients undergoing LT in France are recorded in a national registry ("Cristal") of the French Organ Sharing Organization ("Agence de Biomédecine"). The presence of HCC is identified by a specific code for primary, secondary or tertiary indication, and reported at the time of listing. We considered all patients who underwent LT for or with HCC in France between 01/01/2008 and 31/12/2020.

Inclusion criteria were age ≥18 years and HCC recurrence after LT between 1 January 2008 and 31 December 2021. Exclusion criteria were evidence of a tumour other than HCC (cholangiocarcinoma, combined hepatocellular–cholangiocarcinoma, or another tumour type) on explant pathology and/or at recurrence, and missing data on the date of recurrence, treatment received, or vital status.

Recurrence of HCC was defined either by pathological confirmation through biopsy and/or resection, or by radiological criteria according to EASL Guidelines in patients with recurrent liver disease, and confirmed by a multidisciplinary tumour board. Recurrence of HCC was identified by individual analysis of the medical records of all transplanted patients for the Lille, Bordeaux, Paul-Brousse, Beaujon, La Pitié-Salpêtrière, Montpellier, Toulouse and Grenoble centres, or by reported HCC recurrence in the *Cristal* database for the Mondor, Tours, Strasbourg, Marseille, Rennes, Lyon Croix-Rousse, and Lyon Edouard-Herriot centres.

Variables collected

Only considering variables for which >50% of patients had available data, the following data were collected.

On the waitlist: sex, cirrhosis and its risk factors, date of initial HCC diagnosis, bridging therapy (locoregional therapy [transarterial chemoembolization (TACE), percutaneous ablation or external beam radiotherapy (EBRT)] or systemic therapy), AFP kinetics on the LT list (at registration, maximal and last AFP before transplantation), last MELD score before LT.

At LT: age, date of LT, Milan criteria and AFP score, explant pathology (number of nodules, size of largest nodule, presence of necrosis, the highest grade of differentiation among all nodules, according to the WHO classification and microvascular invasion). Because of heterogeneous delays between last imaging and LT, and inconsistent radiological protocols for tumour burden determination across centres, we decided to calculate fulfilment of the Milan criteria and the AFP score using explant tumour burden (Table S1). Because data were missing for >50% of patients, data on perineural invasion or histological subtype of HCC were not analysed in this study.

During post-transplant follow-up: recurrence of liver disease (defined as daily alcohol relapse, recurrence of chronic hepatitis B or C), persistence or onset of obesity, and

immunosuppressive regimen. Because of heterogeneous management between and within centres and because data were missing for >50% of patients, surveillance imaging modalities, the occurrence of allograft fibrosis, or rejection episodes could not be analysed for the purpose of this study.

At HCC recurrence: date of recurrence, organs affected by recurrence, tumour number and largest diameter, ECOG performance status, modification of immunosuppressive regimen upon recurrence, initial treatment applied, classified as "curative intent" (R0 resection or percutaneous ablation) or "non-curative intent" (including locoregional treatments such as TACE and EBRT, systemic treatments) or supportive care only. Patients receiving a combination of curative and non-curative treatments (for example, resection of one lesion and radiotherapy of another) were classified as receiving multimodal treatment.

Statistical analysis

The primary outcome was OS after recurrence of HCC, defined as the time interval between the diagnosis of recurrence and death from any cause or last follow-up.

Secondary outcomes were time to recurrence, defined as the time interval between the date of LT and the diagnosis of recurrence; OS after transplantation, defined as the time interval between the date of LT and death from any cause or last follow-up.

OS was estimated using the Kaplan-Meier method. Factors associated with OS were investigated using a Cox regression model. Among the factors significantly associated with OS in univariable analyses (threshold p <0.05), the most relevant variables with rates of missing data <10% were introduced into a multivariable Cox model. Hazard ratios (HR) and their 95% CIs were derived from the models as a measure of effect size.

Patients were categorised according to the time from transplantation to recurrence into four groups using the following cut-offs: 6 months, usually corresponding to the first dedicated screening for recurrence; 3 years, corresponding to the end of the surveillance currently recommended by the ILTS; and 5 years, corresponding to the most common definition of cancer remission.

AFP kinetics on the transplant waiting list were categorized. In the absence of consensus in the literature on the definitions of AFP increase or decrease, we applied the same thresholds used in the radiological RECIST criteria. Progressive AFP was defined as a >20% increase from the AFP level at listing to the maximum AFP level and/or the last AFP level before LT; decreasing AFP was defined as a >30% decrease from the maximum AFP level to the last AFP level before LT; stable AFP was defined as neither progressive nor decreasing AFP.

Qualitative variables were described in terms of frequencies and percentages, calculated on patients with available data, considering that data were missing at random. Quantitative variables were described by median (IQR) because of the non-parametric distribution of most variables, as verified using the Shapiro-Wilk test. Qualitative and quantitative variables were compared using the chi-square test, Fisher's exact test and Wilcoxon tests.

To account for potential confounding by indication in the association between curative treatment of recurrence and OS, we performed propensity score matching. The propensity score, defined as the probability of receiving curative treatment for recurrence, conditional on pre-treatment covariates, was estimated using a logistic regression model including the following covariates selected *a priori* based on clinical relevance and expected prognostic value: time to recurrence, total number of recurrence sites, liver recurrence, bone recurrence, and serum AFP levels. Patients were matched 1:1 using nearest-neighbour matching without replacement, with a calliper width of 0.2 standard deviations of the logit of the propensity score, using the MatchIt package.

The significance level was set at 5%. Statistical analyses were performed using R software version 4.5.1.

Results

Pre-transplant characteristics and explant pathology of patients with HCC recurrence

From 1 January 2008 to 31 December 2020, 4,626 adult patients underwent deceased donor LT for HCC in the 15 participating centres. Among them, we identified 451 patients with tumour recurrence diagnosed before December 31, 2021, corresponding to a 9.7% recurrence rate. A total of 63 patients were excluded from the study because of incidental cholangiocarcinoma or mixed hepato-cholangiocarcinoma on explant pathology (n = 54), angiosarcoma on explant pathology (n = 1), cholangiocarcinoma at recurrence despite HCC on explant pathology (n = 6), and missing data (n = 2). In total, 388 patients with HCC recurrence were analysed in the present study (flowchart in Fig. S1).

Characteristics before LT are detailed in Table S2. Patients were mostly male (n = 332, 85.6%), with underlying cirrhosis (n = 367, 94.6%), the median age at transplantation was 60.4 years (IQR 55.2-63.8). The main risk factors for cirrhosis were harmful alcohol consumption (n = 227, 58.5%), chronic hepatitis C (n = 148, 38.1%), and diabetes (n = 112, 29.5%). HCC was diagnosed a median of 19.8 months before LT (IQR 12.5-33.1), and 312 patients (80.4%) underwent bridging therapies before LT, mostly TACE (n = 271, 86.8%). The median last serum AFP before LT was 16 ng/ml (IQR 5-100.5). Regarding AFP evolution on the waiting list (among 355 patients with at least two measurements), 171 patients (48.2%) had stable AFP levels (156 consistently below 100 ng/ml and 15 consistently above 100 ng/ml), 66 (18.6%) had decreasing AFP levels (72% of whom had AFP levels below 100 ng/ml before LT), and 118 (33.2%) had increasing AFP levels (43.9% of whom had AFP levels above 100 ng/ml before LT).

Explant pathology data, available for 378 patients, are detailed in Table S3. Most patients had up to 3 nodules (n = 202, 53.4%), and 27 patients (7.1%) had no sign of viable tumour. Overall, 204 patients had HCC (54%) exceeding Milan criteria on the explant. When considering the last pre-LT AFP and explant pathology, 114 patients (30.1%) exceeded AFP model criteria (score >2).

Heterogeneity of recurrence patterns is associated with patient and tumour characteristics

Patient and tumour characteristics at recurrence are detailed in Table 1. Median time from LT to recurrence was 19.1 months (IQR 10.4-36.5), median age at recurrence was 62.5 years old

Table 1. Characteristics of patients at the time of recurrence of hepatocellular carcinoma after liver transplantation.

	N	
**Time from LT to recurrence (months)**	388	19.1 (10.4-36.5)
**Patient factors at recurrence**		
Age	388	62.5 (57-66.2)
PS >1	347	43 (12.4)
Recurrent liver disease	373	64 (17.2)
HCV recurrence	144*	47 (32.6)
Any alcohol relapse	169*	26 (15.4)
Daily alcohol relapse	169*	13 (7.7)
Obesity	317	76 (24.0)
Diabetes	380	112 (29.5)
**Tumour distribution**		
Organs affected	388	
Liver		152 (39.2)
Lungs		123 (31.7)
Bones		100 (25.8)
Lymph nodes		92 (23.7)
Peritoneal		74 (19.1)
Others		85 (21.9)
Distribution	388	
One organ		240 (61.9)
Two organs		106 (27.3)
Three or more organs		42 (10.8)
Liver-only		77 (19.8)
Extrahepatic-only		236 (60.8)
Both		75 (19.4)

N, number of patients with available data. Quantitative variables are presented as median (IQR), qualitative variables as n (%). Percentages are calculated within each line on patients with available data.

LT, liver transplantation; PS, performance status.

*Specifically indicates that the percentage was calculated among patients who had HCV and/or excessive alcohol consumption before transplantation.

(IQR 57-67.2). In 236 patients (60.8%), recurrent HCC was exclusively extrahepatic at the time of diagnosis. In 75 patients (19.4%), HCC recurred both within and outside the liver, and for the remaining 77 patients (19.8%), HCC recurred in the liver allograft only. Overall, most HCC recurrences after LT (n = 240, 61.9%) occurred only in one organ, either in the liver (n = 77, 19.8%); the lungs (n = 43, n = 11.1%), the bones (n = 44, n = 11.3%), or lymph nodes (n = 31, 10.8%).

The anatomical pattern of lesions according to time to recurrence is detailed in Fig. 1A and Table 2. Forty-eight patients (12.4%) had recurrence within 6 months after LT, 238 (61.3%) between 6-36 months, 62 (16%) between 36 and 60 months, and 40 (10.3%) more than 5 years after LT. Very early recurrences (within 6 months after LT) mainly occurred in the lungs (43.8%), bones (37.5%), liver and lymph nodes (29.2% each). Very late recurrences (after 60 months) were mostly liver-confined disease with less frequent extrahepatic involvement. Median time to recurrence for extrahepatic-only *vs*. hepatic-only *vs*. both intra- and extrahepatic recurrences were 17.6 *vs.* 22.9 *vs.* 15.8 months, respectively (p <0.001). Single-organ disease appeared to be more frequent in later recurrences, from 52.1% for very early recurrences to 72.5% for very late recurrences, without statistical significance (p = 0.25).

Time to recurrence was associated with tumour-related factors (Fig. 1B and Table 2). Twenty (44.4%) and 29 (64.4%) patients presenting with early recurrence (<6 months) exceeded AFP and Milan criteria, respectively. Patients exceeding Milan criteria had shorter median time to recurrence compared to those within Milan criteria (17.9 *vs.* 20.4 months, p = 0.02). The median time to recurrence in patients with AFP model scores of 0, 1, 2 and >2 was 21.8, 21.6, 18.4, and 15.7 months, respectively (p = 0.004). Time to recurrence significantly differed according to tumour evolution on the waiting list. Time to recurrence for patients who received no bridging therapy, LRT-only, *vs.* systemic therapy were 25.3 *vs.* 17.6 *vs.* 14.2 months (p <0.001). Patients with stable AFP *vs.* decreasing AFP *vs.* increasing AFP had median time to recurrence of 20.5 *vs.* 19.7 *vs.* 16.6 months (p = 0.03).

Patient-related factors were also associated with time to recurrence. Recurrence of chronic liver disease in the liver graft was more frequent among patients with later HCC recurrence (p <0.001), with more frequent HCV recurrence (p <0.001), more frequent alcohol relapse (p = 0.02), more frequent grade 2 obesity (p = 0.03), and a non-significant trend towards more frequent diabetes (p = 0.18) (Fig. 1C and Table 2).

Surgical and ablative treatments are associated with the best outcomes in recurrent HCC after LT

Patients were treated with surgical resection and/or percutaneous ablation of all lesions (n = 99 including 79 resections, 17 ablations and 3 both, 25.5%), LRT only (TACE and/or EBRT, n = 30, 7.7%), a combination thereof (n = 25, 6.4%, mostly surgery and radiotherapy), systemic treatment (n = 188, 48.5%), or best supportive care (BSC, n = 45, 11.6%).

The use of surgical/ablative treatment varied according to the time to recurrence (Fig. 2A). Systemic treatment (n = 25/48, 52.1%) and BSC (n = 14/48, 29.2%) were the main options in very early (<6 months) recurrence. Surgical/ablative treatments were the most frequently used options for recurrences occurring between 36-60 months (28/62, 45.2%) and after 60 months (13/40, 32.5%).

Median overall post-recurrence survival was 18.3 months (95% CI 15.6-21.3). 1-, 3-, and 5-year OS rates after recurrence were 62.5% (95% CI 57.8-67.5), 27.4% (23.1-32.5), and 12.7% (95% CI 9.2-17.4), respectively. Median OS differed significantly according to treatment group (surgery/ablation, combined treatment, locoregional treatment, systemic treatment or BSC) and was 42.2 (31.9-58.7), 28.9 (23.2-NR), 14 (12.1-32.6), 14.7 (12-17.2), or 2.2 (1.9-3.9) months, respectively (p <0.001, Fig. 2B).

Median overall post-LT survival was 43.6 months (95% CI 40.6-49.9). 5- and 10-year post-LT OS for patients who developed HCC recurrence after LT were 38.8% (95% CI 34.2-44.1), and 11.6% (95% CI 8.3-16.3), respectively. OS significantly differed after surgery/ablation, combined, locoregional, systemic, or BSC (p <0.001, Fig. 2C), with median OS of 82.2 (70.6-95.8), 60.6 (43.7-NR), 46.2 (31.7-71.7), 36.4 (32.2-41.6), or 15.6 (12.6-27.2) months, respectively. For patients with a recurrent HCC after LT amenable to a surgical or ablative treatment with curative intent, 5- and 10-year OS rates after transplantation were 67.0% (95% CI 58.1-77.3) and 26.4% (95% CI 17.1-40.7), respectively.

Tumour burden at recurrence in each treatment group is detailed in Table S4, showing that surgery/ablation was applied mostly for patients with only one organ involved (92/99, 93%). Overall, surgery/ablation was performed in 39/152 (25.7%) patients with liver, 15/123 (12.2%) with lung, 9/100 (9%) with bone, 15/92 (16.3%) with lymph node, 14/68 (20.6%) with peritoneal/parietal, 11/29 (37.9%) with adrenal

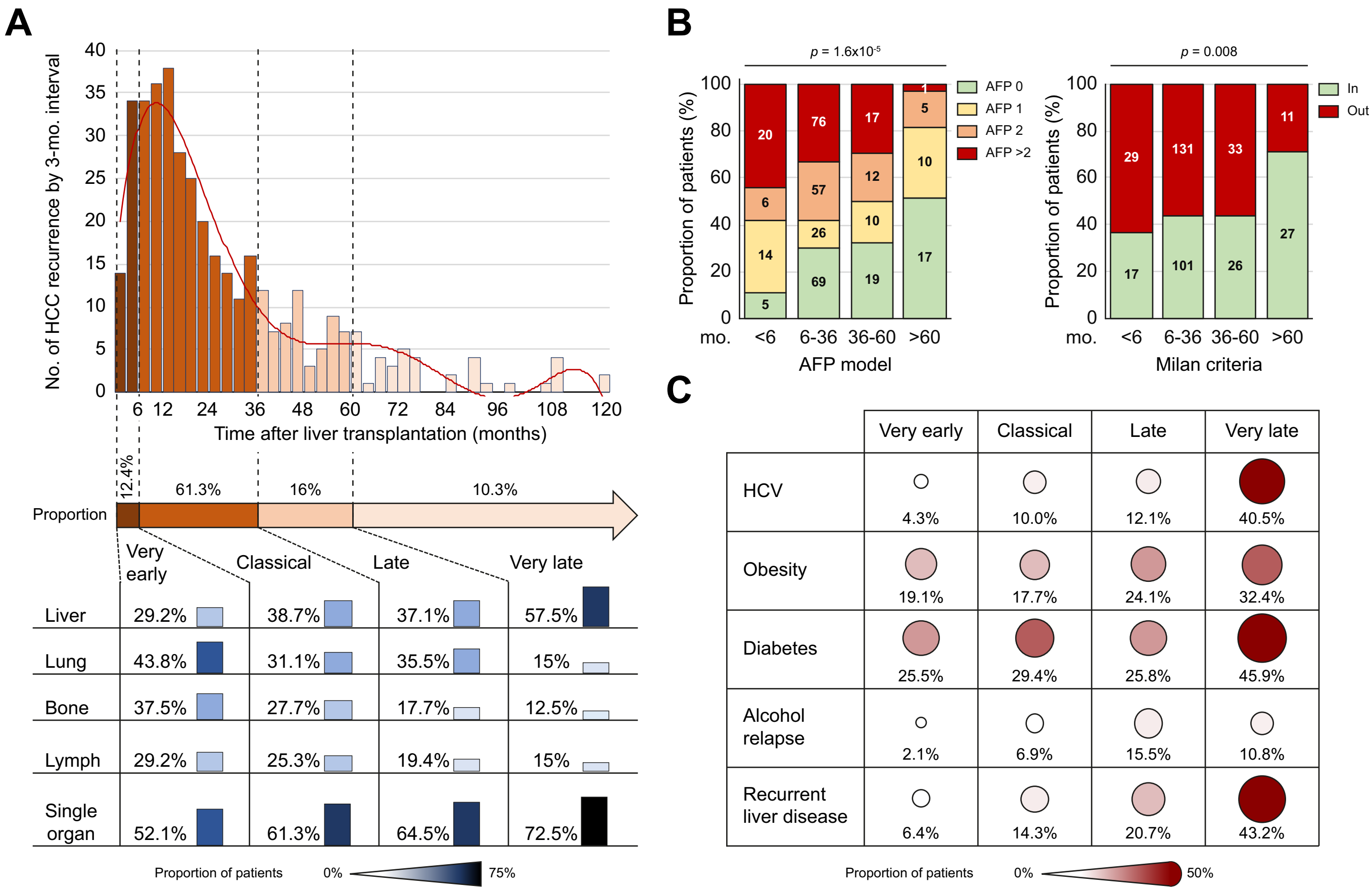

Fig. 1. Heterogeneity of recurrence patterns associate with tumour and patient characteristics (A) Density distribution of recurrent hepatocellular carcinoma according to time after transplantation (red line represents a polynomial function with 6 degrees of freedom, reported as the best fit, R^2 = 0.9323). Patients are subsequently categorized into 4 groups of delay to recurrence, each associated below to the anatomical distribution of recurrent lesions, presented as proportion among each group of delay. (B) Distribution of patients with tumours according to AFP model (left) and Milan criteria (right) by subgroups of recurrence delay, comparisons and *p* values using χ^2 test, level of significance set at $p \leq 0.05$. (C) Proportions of patients presenting with recurrent risk factor and liver disease after transplantation according to delay of recurrence. AFP, alpha-fetoprotein; HCC, hepatocellular carcinoma.

recurrence, and 16/55 (29.1%) with other sites of recurrence. Surgery/ablation was the most frequent option for patients with liver-confined recurrence (35/77, 45.5%), while proposed in 60/235 (25.5%) of patients with extrahepatic-only disease.

Considering the imbalance in tumour burden and time to recurrence between patients treated with curative *vs.* non-curative approaches, we performed an additional comparison after matching using a propensity score. The covariates used for the construction of the propensity score were time to recurrence, the number of organs affected by recurrence (1 or >1), the presence of liver lesions, the presence of bone lesions, and AFP levels at recurrence, factors that may have influenced treatment allocation. After matching (covariate balance in Fig. S2), 75 patients treated with curative intent were compared with 75 matched patients treated with non-curative intent. OS was better for patients treated with a curative approach compared to a non-curative approach (*p* <0.0001, Fig. 2D).

When focusing on a subgroup of interest of 218 patients presenting with only one organ affected by recurrence, excluding patients for whom BSC was the only applicable option (tumour burden in this subgroup detailed in Table S5), median OS still significantly favoured a curative approach compared to a non-curative approach (42.2 [31.9-58.7] *vs.* 19.2 [15.5-26.1] months, respectively, *p* <0.001, Fig. S3A). In this subgroup of interest, surgery/ablation was consistently associated with better OS than systemic treatments for liver-only (HR 0.4, 0.21-0.71, *p* = 0.002), lung-only (HR 0.34, 0.13-0.86, *p* = 0.02) and lymph-node-only (HR 0.33, 0.14-0.81, *p* = 0.02) disease (Fig. S3B). Due to small sample sizes (<10 patients in each group), the benefit of radical approaches for bone-only, peritoneal-only, adrenal-only or other extrahepatic localizations could not be formally tested.

Among patients who underwent surgical resection and/or ablation of all lesions (n = 99), 71 patients presented with new recurrence with a median time to new recurrence of 9.2 months (IQR 4.2-16.5), 22 patients remained free of any new recurrence with a median follow-up of 34.3 months (IQR 18.2-51.9), 6 had missing data. The 3-year recurrence-free survival rate was 25.8% (95% CI 18-37.1%).

Outcomes of recurrent HCC after LT following the advent of new systemic treatments

A total of 283 patients received systemic treatment, either as a primary treatment (n = 188) or after failure of surgery/ablation/

Table 2. Characteristics of recurrent hepatocellular carcinoma according to the time to recurrence.

	N	Early (<6 months)	Classical (6-36 months)	Late (36-60 months)	Very late (>60 months)	p values
		48	238	62	40	
Organs	388					
Liver		14 (29.2)	92 (38.7)	23 (37.1)	23 (57.5)	0.05
Lungs		21 (43.8)	74 (31.1)	22 (35.5)	6 (15)	0.03
Bones		18 (37.5)	66 (27.7)	11 (17.7)	5 (12.5)	0.02
Lymph nodes		14 (29.2)	60 (25.3)	12 (19.4)	6 (15)	n.t.
Adrenal		5 (10.4)	18 (7.6)	4 (6.5)	2 (5)	n.t.
Peritoneal/seeding		10 (20.8)	46 (19.3)	11 (17.7)	7 (17.5)	n.t.
Others		9 (18.8)	28 (11.8)	13 (21)	6 (15)	n.t.
Distribution	388					0.008
Liver-only		2 (4.2)	46 (19.4)	14 (22.6)	15 (37.5)	
Extrahepatic-only		34 (70.8)	146 (61.6)	39 (62.9)	17 (42.5)	
Both		12 (25)	46 (19.4)	9 (14.5)	8 (20)	
Number of organs	388					
Single organ		25 (52.1)	146 (61.3)	40 (64.5)	29 (72.5)	0.25
Primary HCC						
No bridging	388	3 (6.3)	43 (18.1)	16 (25.8)	14 (35)	0.004
TACE bridging	388	40 (83.3)	173 (72.7)	39 (65.0)	19 (47.5)	n.t.
PA bridging	388	5 (10.4)	32 (13.4)	8 (12.9)	6 (15)	n.t.
ERT bridging	388	5 (10.4)	12 (5.0)	1 (1.6)	0	n.t.
Systemic-bridging	388	5 (10.4)	15 (6.3)	1 (1.6)	0	n.t.
Milan-Out	375	29 (63.0)	131 (56.5)	33 (55.9)	11 (28.9)	0.008
AFP Model-Out	364	20 (44.4)	76 (33.3)	17 (29.3)	1 (3.0)	$1.6x10^{-5}$
Patient factors post-LT						
Recurrent liver disease	373	3 (6.4)	33 (14.3)	12 (20.7)	16 (43.2)	$3.3x10^{-5}$
HCV recurrence	144*	2 (4.3)	23 (10.0)	7 (12.1)	15 (40.5)	$3.7x10^{-6}$
Daily alcohol relapse	169*	1 (2.1)	16 (6.9)	9 (15.5)	4 (10.8)	0.11
Obesity	317	9 (19.1)	41 (17.7)	14 (24.1)	12 (32.4)	0.23
Diabetes	380	12 (25.5)	68 (29.4)	15 (25.8)	17 (45.9)	0.19

N, patients with available data. n.t., not tested.
Quantitative variables are presented as median (IQR), qualitative variables as n (%). Percentage on patients with available data within each category of time to recurrence. Comparisons and p values using χ^2 test, level of significance set at $p \leq 0.05$. ERT, external radiotherapy; HCC, hepatocellular carcinoma; LRT, locoregional therapy; LT, liver transplantation; PA, percutaneous ablation; TACE, transarterial chemoembolization.
*Indicates the number of patients with pretransplant HCV and/or excessive alcohol consumption and available data on recurrence of liver disease.

locoregional treatments (n = 95). Median OS from the time of first-line systemic treatment initiation was 13.4 months (95% CI 11.4-16.2). Two, three, or more lines of systemic therapies were used in 150 (53%), 70 (24.7%), and 34 (12%) patients. The type and lines of systemic treatments are detailed in Table S6. Sorafenib was the most frequent first-line systemic treatment overall (n = 232, 82%), followed by lenvatinib (n = 28, 7%). Immunotherapy was used in 16 patients (3, 3, 2, and 8 patients as first-, second-, third-, and further-line therapy, respectively).

The earliest use of regorafenib was in May 2016 and the earliest use of lenvatinib was in July 2018. Therefore, we compared outcomes between patients across three eras according to the date of recurrence: 2008-2015 (n = 133), 2016-2017 (n = 109), and after 2018 (n = 146). Patient characteristics and treatments differed across the three eras in terms of time to recurrence (with more late recurrences in patients after 2018, p = 0.0007), but were similar in the use of curative approaches. After 2016, regorafenib became the most-frequent second-line treatment (59.2% for 2016-2017, 41.0% after 2018). After 2018, sorafenib (n = 71, 70.3%), and lenvatinib (n = 24, 23.8%) were the most frequent first-line treatment options. Access to first-, second-, and third-line treatments was not statistically different across periods (Table S7).

When considering all patients (n = 388) according to the date of recurrence diagnosis, there was no significant improvement between patients diagnosed before 2016, between 2016 and 2017, or after 2018 (p = 0.09, Fig. 3A), with median OS of 17.5 *vs.* 15.1 *vs.* 20.0 months, respectively. The difference remained statistically non-significant after adjustment for the time to recurrence (HR 1.14 for 2016-2017 *vs.* 2008-2015, 95% CI 0.87-1.49, p = 0.34; HR 0.88 after 2018 *vs.* 2008-2015, 95% CI 0.67-1.17, p = 0.39). There was also no significant improvement in OS among patients starting systemic therapy (n = 277, 6 patients with no follow-up data excluded) across dates of initiation (p = 0.17, Fig. 3B).

When focusing on patients diagnosed and starting first-line systemic therapy after 2018 when both sorafenib and lenvatinib were available in France, we observed significantly longer survival with lenvatinib compared to sorafenib (p = 0.03, Fig. 3C), with a median OS of 29.2 *vs*. 15.8 months, respectively. However, the characteristics of the two groups were imbalanced (Table S8). Compared with sorafenib, lenvatinib was more frequently used after initial treatment with curative intent and for a new recurrence (37.5% *vs*. 9.9%, respectively; p = 0.005), was initiated later after the diagnosis of recurrence (6.5 *vs*. 2 months; p = 0.008), and was used in patients with a lower tumour burden (>1 organ affected in 20.8% *vs.* 53.5%,

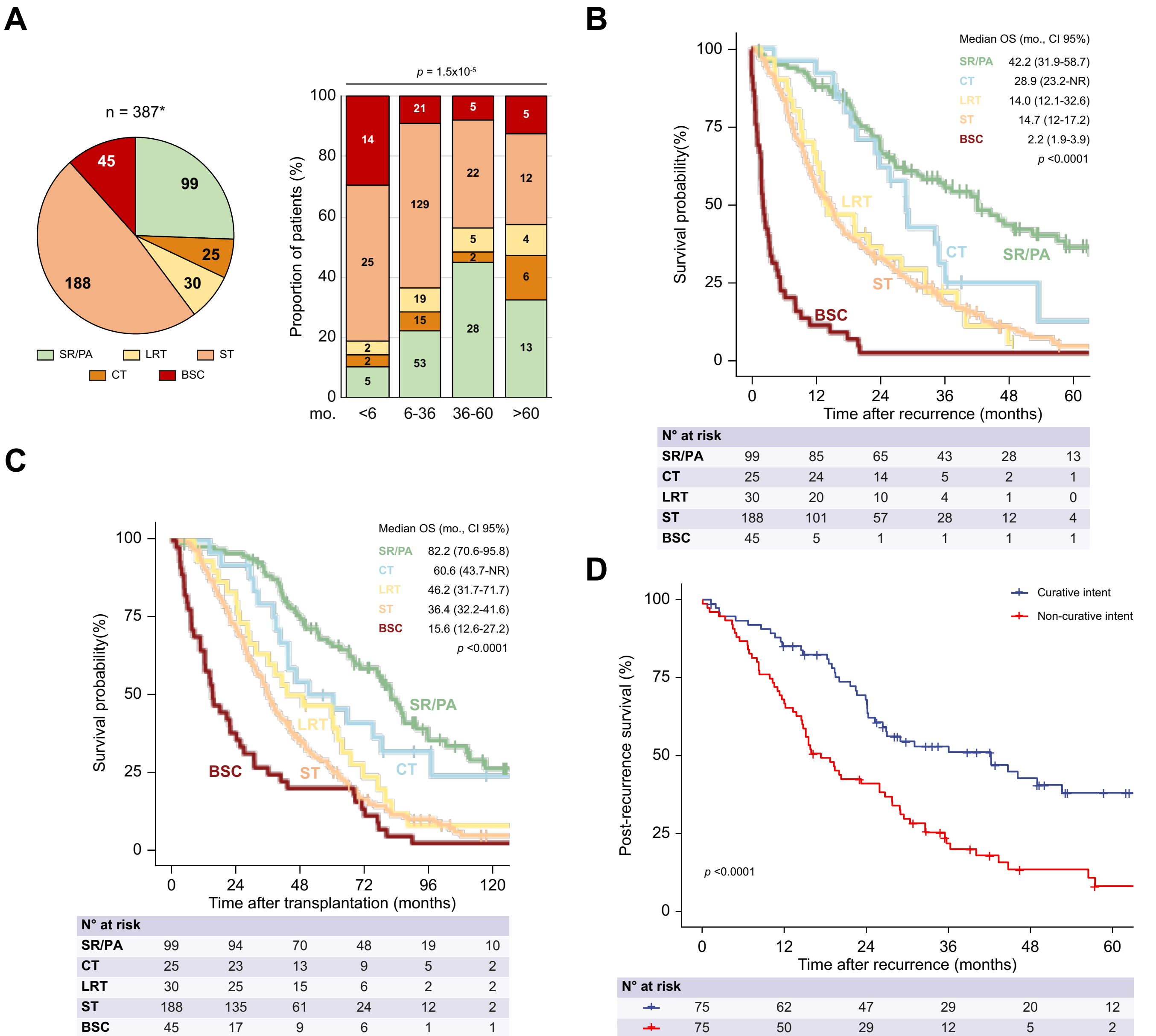

Fig. 2. Surgical and ablative treatments of all extrahepatic and/or intrahepatic lesions are associated with the best outcomes in recurrent hepatocellular carcinoma after liver transplantation First treatment applied to patients with recurrent hepatocellular carcinoma after liver transplantation (*, excluding 1 patient without data on treatment), overall (left) and by recurrence delay (right) compared with the χ^2 test. (B) Post-recurrence and (C) post-transplantation overall survival of patients according to the first treatment applied, compared with the log-rank test. (D) Post-recurrence survival using curative *vs.* non-curative approach after propensity score matching. Level of significance set at $p \leq 0.05$. n, number of patients with available data. SR, surgical resection. BSC, best supportive care alone; CT, combined ablative/surgical and locoregional non-ablative treatment; HR, hazard ratio; LRT, non-ablative locoregional treatment; Mo., months; NR, not reached; OS, overall survival; PA, percutaneous ablation; p-r., post-recurrence; SR, surgical resection; ST, systemic therapy.

respectively; p = 0.009) and less frequent liver recurrence (12.5% *vs*. 40.8%, respectively; p = 0.02). Time to recurrence did not differ significantly between the groups.

The impact of extended selection criteria in France on outcomes in patients with recurrent HCC after LT

The main characteristics of patients according to the era of selection criteria (pre-*vs.* post-AFP model) are described in Table 3. Pre-transplant characteristics were similar between eras, except time from HCC diagnosis to LT which was longer after AFP model implementation (20.8 *vs.* 17.1 months, p = 0.005). Tumour burden and microvascular invasion on explant pathology did not differ. Explant tumour burden outside Milan criteria did not differ before *vs.* after the adoption of the AFP model (53.6 *vs.* 54.7%, p = 0.93). The distribution of AFP score before and after adoption of the AFP model is detailed in Fig. S4.

Patients transplanted before AFP model implementation had a longer time to recurrence (31.3 *vs.* 17.0 months, p <0.001), with later recurrences more prevalent among patients transplanted before the adoption of the AFP model (Fig. 4A).

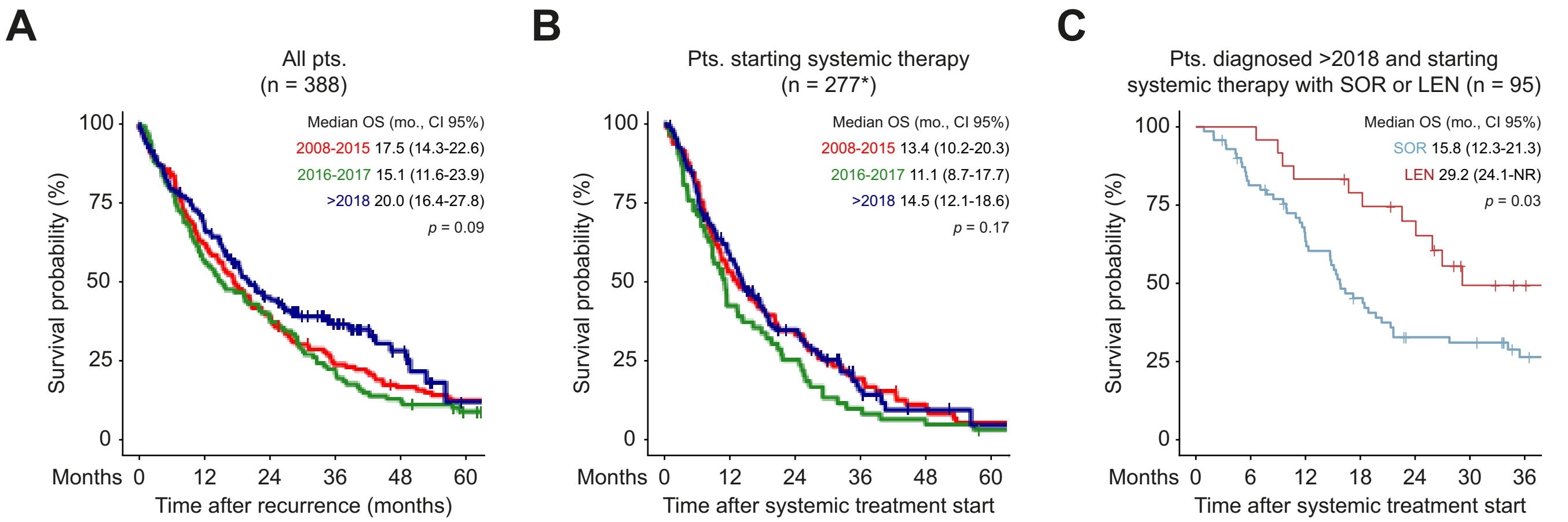

A — N° at risk

N° at risk	0	12	24	36	48	60
2008-2015 (red)	133	82	52	30	20	15
2016-2017 (green)	109	61	41	24	13	6
>2018 (blue)	146	93	55	27	11	1

B — N° at risk

N° at risk	0	12	24	36	48	60
2008-2015 (red)	82	43	27	15	7	4
2016-2017 (green)	62	25	15	6	3	1
>2018 (blue)	133	72	34	11	3	1

C — N° at risk

N° at risk	0	6	12	18	24	30	36
SOR (light blue)	71	56	42	29	19	18	11
LEN (red)	24	24	20	18	15	8	6

Fig. 3. Outcomes of patients with recurrent hepatocellular carcinoma after liver transplantation from 2008 to 2020 Post-recurrence overall survival comparing of (A) all patients receiving a diagnosis of recurrence and of (B) patients initiating a systemic treatment between 2008 and 2015 (before the approval of regorafenib), 2016 and 2017 (before the approval of lenvatinib and cabozantinib), and after 2018 (all options available), compared with the log-rank test (*, excluding 6 patients with no follow-up data). (C) Overall survival of patients diagnosed after 2018 and initiating a first-line systemic treatment either with SOR (blue) or LEN (red), compared with the log-rank test. Level of significance set at p ≤0.05. n, number of patients with available data. LEN, lenvatinib; NR, not reached; OS, overall survival; Pts, patients; SOR, sorafenib.

When comparing patients according to transplantation performed after *vs.* before the adoption of the AFP model, post-recurrence survival did not significantly differ (p = 0.32, Fig. 4B). Median post-recurrence survival was 17.3 (14.6-21.3) *vs.* 20.3 months (95% CI 15.8-25.2), respectively. The 5-year survival rate was 11.7% (7.7-17.7) *vs.* 15.3% (95% CI 9.6-24.6).

Patients with HCC recurrence transplanted after the AFP model adoption had worse 5-year and 10-year post-LT survival *vs.* patients transplanted before (30.4% *vs.* 57.2%, and 10.6% *vs.* 16.2%, respectively, Fig. 4C, $p = 3x10^{-4}$). However, when adjusting for the time to recurrence, the difference was no longer significant (HR 1.02, 95% CI 0.79-1.32, p = 0.9). Survival curves stratified by the time to recurrence are presented in Fig. 4D.

To explore the possibility of a survivorship bias associated with longer post-LT follow-up for patients transplanted before AFP model adoption (allowing more time for later recurrences to be detected), we performed a sensitivity analysis including only patients receiving LT until 2016 (to allow for at least a 5-year window for all patients), and focusing only on recurrences within the first 60 months after LT. Again, patients transplanted after AFP model adoption had earlier recurrences (p = 0.02, Table S9).

To further evaluate the impact of tumour characteristics on survival after recurrence, we analysed the association of pretransplant risk-stratifying scores and their individual components with post-recurrence survival. Adherence to both the Milan criteria and the AFP model was significantly associated with survival after recurrence (Fig. S5A). Post-recurrence survival also significantly differed according to tumour size, the number of nodules on explant pathology, microvascular invasion, and last serum AFP levels before LT, without significant difference according to tumour differentiation (Fig. S5B).

The impact of immunosuppressive regimen on outcomes of recurrent HCC after LT

We analysed data from 370 patients with available information on immunosuppressive regimens before and after the diagnosis of recurrent HCC following LT. In 104 patients (28.1%), mTOR inhibitors (mTORi, sirolimus or everolimus) were introduced before recurrence, at a median of 2.6 months (IQR 1.6-7) after LT, and 14.9 months before recurrence (IQR 6-26.1). Tumour characteristics differed significantly between patients who received mTORi before recurrence and those who did not. Patients who received pre-recurrence mTORi had higher maximal and last AFP levels before LT, more nodules on explant, and were more frequently outside the Milan criteria and had an AFP score >2 (Table S10).

Then, we analysed patients according to immunosuppressive management at the diagnosis of recurrence. After the diagnosis of HCC recurrence, 188 patients (63.7% of 295 with available data) continued to receive a calcineurin inhibitor (CNI), 127 (43.3% of 293) continued to receive mycophenolate mofetil (MMF), and 257 (69.5% of 370) received an mTORi as part of their post-recurrence immunosuppressive regimen. We analysed 293 patients with available data both on the timing of mTORi introduction and of CNI management (Table S11). The introduction of mTORi and CNI management were associated: CNIs were maintained in almost all patients who did not receive mTORi before or at recurrence, whereas CNIs were discontinued in 104/196 patients (53.1%) who received mTORi before or at recurrence. For patients with data available for all immunosuppressive drugs (n = 270), 74 (26.6%), 199 (71.6%), 5 (1.8%) patients had 1, 2 or 3 drugs in their regimen after the diagnosis of recurrence. There was no difference in post-recurrence survival between patients who received one or two immunosuppressive drugs (p = 0.71, median OS 18.5 *vs.*

19.4 months, Fig. 5A). There was no difference in post-recurrence survival between patients who remained on a CNI or not (median OS 18.2 *vs.* 21.6 months, p = 0.1, Fig. 5B). There was no difference in post-recurrence survival between patients who remained on MMF or not (median OS 17.3 *vs.* 21.6 months, p = 0.4, Fig. 5C).

Time to recurrence was 2.5 months shorter in patients receiving mTORi (p = 0.05). No significant differences were observed in tumour distribution or access to curative-intent treatment between patients with and without mTORi in their post-recurrence immunosuppressive regimen (Table S12). However, we observed a significantly longer post-recurrence survival in patients who received an mTOR inhibitor in their post-recurrence regimen compared to those who did not (median OS 20.7 *vs.* 13.2 months, p = 0.001, Fig. 5D). This difference was observed both in patients who underwent a curative treatment (median OS 52.6 *vs.* 25.2 months, p = 0.01, Fig. 5E) and in those who were treated with a non-curative treatment (median OS 15.4 *vs.* 9.3 months, p = 0.03, Fig. 5F).

Overall, in a multivariable Cox regression, time to recurrence (for 6-36 months, HR 0.59, 95% CI 0.41-0.84, p = 0.004; for 36-60 months, HR 0.44, 95% CI 0.27-0.69, p <0.001; for >60 months, HR 0.34, 95% CI 0.19-0.6, p <0.001), having more than one organ affected by recurrence (HR 1.36, 95% CI 1.04-1.78, p = 0.03), the presence of liver lesions (HR 1.56, 95% CI 1.19-2.04, p = 0.001), the presence of bone lesions (HR 1.50, 95% CI 1.14-1.98, p = 0.004), treatment with curative intent (HR 0.43, 95% CI 0.31-0.6, p <0.001), and mTORi (HR 0.62, 95% CI 0.48-0.79, p <0.001) all remained significantly associated with post-recurrence mortality (Table S13).

Table 3. Characteristics of patients according to era of transplant selection model.

Selection era for LT	Before AFP model	After AFP model	
**N**	**117**	**271**	***p* values**
**Pre-transplantation**			
Months between HCC diagnosis to LT	17.1 (9.4-25.9)	20.8 (14.1-34.6)	**0.005**
Any bridging therapy	91 (77.8)	221 (81.5)	0.47
AFP at listing	9.5 (5-32)	8.1 (4.1-32)	0.55
Maximal AFP	20 (6-78)	16.6 (6-86.5)	0.97
Last AFP pre-LT	19 (5-87)	16 (5-115)	0.84
AFP dynamics on waiting list			0.21
Stable AFP	51 (56.0)	120 (45.5)	
Decreasing AFP >30%	15 (16.5)	51 (19.3)	
Increasing AFP >20%	25 (27.5)	93 (35.2)	
**Explant characteristics**			
Number of nodules			0.77
0 (pathological complete response)	7 (6.3)	20 (7.5)	
1-3	58 (51.8)	144 (54.1)	
4+	47 (42.0)	102 (38.3)	
Size of the largest nodule			0.48
<3 cm	55 (51.4)	150 (57.0)	
3-6 cm	45 (42.1)	93 (35.4)	
>6 cm	7 (6.5)	20 (7.6)	
Microvascular invasion	55 (52.9)	136 (53.1)	1
**Adherence to selection criteria using explant tumour burden**			
Outside Milan criteria	59 (53.6)	145 (54.7)	0.93
AFP model >2	35 (34.7)	79 (30)	0.47
**Time to recurrence (months)**	31.3 (12.1-57.7)	17.0 (10.1-30.6)	**2×10^{-6}**
<6 months	13 (11.1)	35 (12.9)	**5×10^{-10}**
6-36 months	48 (41.0)	190 (70.1)	
36-60 months	29 (24.8)	33 (12.2)	
>60 months	27 (23.1)	13 (4.8)	
**Recurrent tumour distribution**			
Liver	48 (41.0)	104 (38.4)	0.71
Lungs	34 (29.1)	89 (32.8)	0.52
Bones	26 (22.2)	74 (27.3)	0.34
Number of organs			0.08
One	82 (70.1)	157 (58.1)	
Two	27 (23.1)	79 (29.3)	
Three or more	8 (6.8)	34 (12.6)	
Distribution			0.56
Liver-only	27 (23.1)	50 (18.5)	
Extrahepatic-only	69 (59.0)	167 (61.6)	
Both	21 (17.9	54 (19.9)	
**1st treatment for recurrence**			0.37
Curative approach	34 (29.1)	65 (24.0)	
Non-curative approach	83 (70.9)	205 (75.6)	

N, number of patients with available data. Quantitative variables are presented as median (IQR), qualitative variables as n (%). Percentages are calculated within each column on patients with available data. Comparisons and p values using χ^2 test for qualitative variables, and Wilcoxon rank-sum test (*i.e.* Mann-Whitney *U* test) for quantitative variables, level of significance set at p ≤0.05. AFP, alpha-fetoprotein; HCC, hepatocellular carcinoma; LT, liver transplantation.

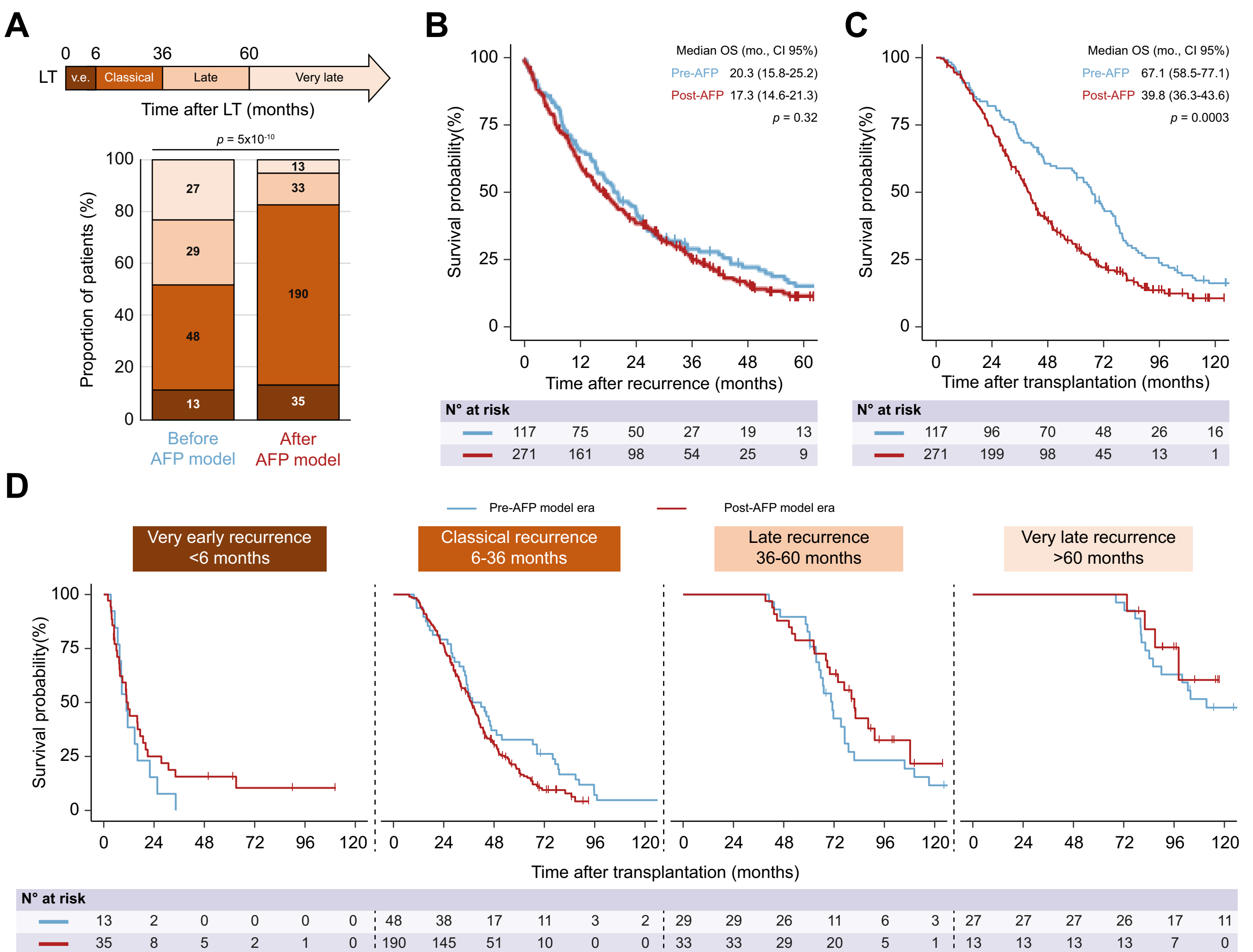

Fig. 4. The impact of extended criteria implementation on outcomes of patients with recurrent hepatocellular carcinoma after liver transplantation (A) Distribution of recurrence delay according to the era of liver transplantation, of patients receiving transplantation before *vs.* after the implementation of the AFP model in France, compared with the χ^2 test. (B) Post-recurrence and (C-D) post-transplantation overall survival of all patients (B-C) and by subgroup of recurrence delay (D), receiving transplantation before (blue) *vs.* after (red) the implementation of the AFP model, compared with log-rank test. Level of significance set at $p \leq 0.05$. n, number of patients with available data. AFP, alpha-fetoprotein; LT, liver transplantation; OS, overall survival; V.e, very early.

Discussion

This nationwide study, in a homogeneous system of patient selection and graft allocation, covering two selection eras, highlights the heterogeneity of recurrent HCC after LT. Although most recurrences occurred during the currently recommended surveillance window of 3 years, approximately 30% of HCC recurrences occur beyond that threshold, and 1 in 6 recurrences occurred more than 5 years after transplantation, highlighting a potential underestimation of a persistent long-term post-transplant risk. Risk factors for recurrence include tumour-related factors, but late recurrence was associated with patient-related factors that are known risk factors for *de novo* cirrhosis and HCC. The implementation of extended selection criteria, namely the AFP model, was associated with earlier recurrences. Once HCC has developed, median survival after recurrence mirrors that of advanced HCC in non-transplanted patients and is significantly improved only when aggressive treatment strategies can be applied, suggesting the potential benefit of more intensive surveillance modalities than those currently recommended. The survival benefit of second-generation TKIs observed in non-transplanted patients with advanced HCC does not appear to extend to patients with recurrent HCC after LT. However, our study suggests a potential beneficial effect of mTORi for the treatment of recurrent HCC after LT, which should be prospectively evaluated.

A better and more up-to-date understanding of the natural history of recurrent post-transplant HCC is essential to improve outcomes of a disease that still matches in our cohort the poor prognosis of advanced HCC in non-transplanted patients (with an estimated OS of 18 months). In this context, the applicability of aggressive surgical or ablative approaches for recurrence appeared to be the only major treatment-related disease modifier. Thus, early detection of recurrence through optimized surveillance appears critical. Currently, the 2024 ILTS/ILCA consensus conference recommends a 3-year surveillance window.[6] Our study confirms that the peak incidence

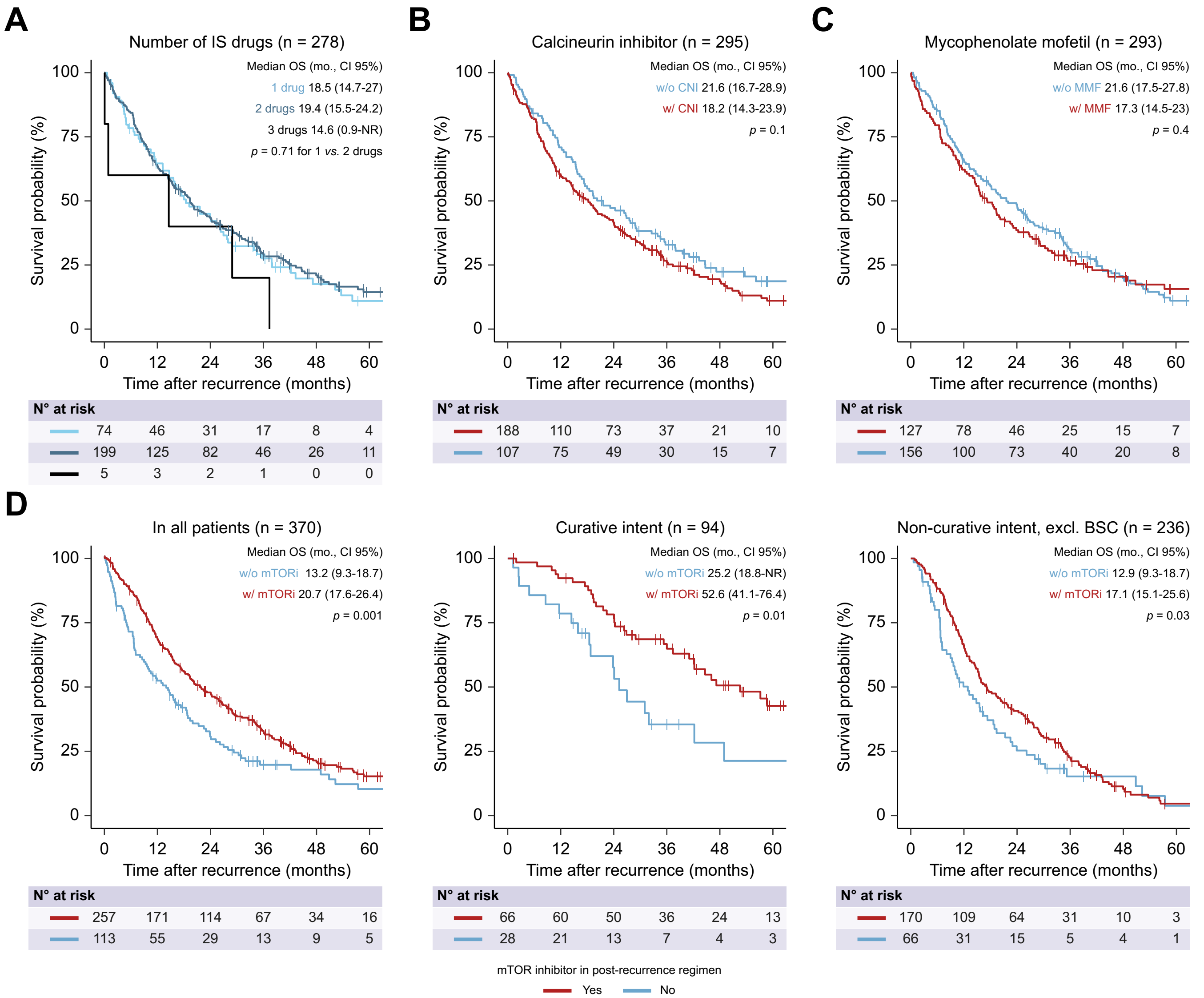

Fig. 5. Immunosuppressive regimen impact on survival of patients with recurrent HCC after transplantation Post-recurrence overall survival according to (A) the number of IS drugs in their regimen, the maintenance of (B) CNIs and/or (C) MMF and/or (D) motoric while treated for recurrence of hepatocellular carcinoma after liver transplantation, comparisons with the log-rank test. Level of significance set at $p \leq 0.05$. n, number of patients with available data. CNIs, calcineurin inhibitors; HCC, hepatocellular carcinoma; IS, immunosuppressive; MMF, mycophenolate mofetil; mTORi, mTOR inhibitor; OS, overall survival.

of recurrence occurs within this window, mainly associated with negative pre-LT tumour characteristics. These recurrences seem to be related to failure of patient selection, owing to pitfalls in radiological/pathological correlation, the lack of integration of the dynamic components of HCC evolution on the waiting list, and the lack of standardized extrahepatic disease monitoring before LT. They can be predicted by adequate post-transplant risk stratification models such as the RETREAT score,[7] the post-MORAL[8] or the R3-AFP[9] score. However, we also observed that one-third of recurrences occurred beyond this window, and one-sixth beyond 5 years post-transplantation. Therefore, models should not only predict recurrence as a binary outcome, but also better distinguish three recurrence profiles: early recurrence (within the first year), in patients who were likely poor candidates for LT, in whom curative therapeutic strategies are less likely to be applicable (more bone recurrence, more multi-organ recurrence, etc ….) and for whom the benefit of monitoring could paradoxically be limited; classical recurrences, for which the application of aggressive surveillance and treatment strategies (including resections of extrahepatic metastases) will be the main determinant of acceptable 10-year post-transplant survival; late recurrences, for which individualized assessment of residual risk, through the screening of recurrent liver disease or identification of immunosenescence, should guide decisions on long-term surveillance, with modalities to be determined by dedicated cost-effectiveness studies. Collectively, our study and others[10] suggest that more accurate post-transplant risk stratification tools will need to account for time-dependent recurrence risk.

The last 15 years have seen a significant push to extend selection criteria for LT in patients with HCC.[3] An important unanswered question was whether those still affected by HCC recurrence also had similar outcomes. The main finding of this

study was that post-recurrence survival did not worsen, but that earlier recurrences appeared more frequent in patients transplanted since the implementation of such extended criteria in France, raising concerns about post-transplant survival in patients with recurrence. It is important to note that the time to recurrence was the only significant difference between patients transplanted before *vs.* after AFP model implementation, without any difference in clinical presentation at diagnosis or treatment application. It can be hypothesized that patients transplanted the most recently also had a shorter follow-up period after transplantation, and that late recurrences may not have occurred at the time of data collection (survivorship bias). However, late recurrences in our study were frequently associated with recurring liver disease. In the modern era, the risk of HCV reinfection, the most frequent factor associated with late recurrence in our study, is now strongly reduced by direct-acting antivirals.[11,12] Whether the increasing prevalence of metabolic risk factors in transplant recipients, as observed in this cohort of patients with HCC recurrence, will also lead to late recurrences is unknown. Longer follow-up is necessary to evaluate how the current epidemiological shifts in transplant candidates are going to impact the incidence of late recurrences, and whether post-transplant outcomes of patients selected in the current extended criteria era will match, or not, those of patients selected before.

In this study, we did not observe any meaningful improvement in survival after HCC recurrence during the last 15 years, further demonstrating the high unmet need in this special population who have been systematically excluded from all pivotal trials of systemic therapy. However, several opportunities emerge from this analysis. First, we observed a statistically significant improvement in survival with lenvatinib compared to sorafenib as a first-line systemic treatment option. However, imbalances in tumour characteristics and the clinical context in which the two drugs were used preclude any definitive conclusions regarding the superiority of lenvatinib. Two other studies have reported a similar signal in the post-transplant setting.[10,13] In the non-transplant advanced HCC setting, the non-inferiority design of the REFLECT trial did not enable demonstration of such superiority.[14] Based on our data, a randomized superiority trial would need to include between 150 and 200 post-transplant patients with recurrent HCC, representing around 1,500 LTs for HCC, assuming a 15% recurrence rate and a 75% systemic treatment rate. Therefore, any prospective trial of systemic treatment for HCC post-transplant will need to be a coordinated international multicentre effort. Second, it is currently unknown whether immunotherapies, as single agents or in combination, have the potential to transform the prognosis of post-transplant recurrent HCC. A major challenge in this setting is the risk of acute rejection. To date, studies have focused on strategies to predict and mitigate such risk, notably by proposing enhanced immunosuppressive regimens.[15] However, keeping in mind the dismal prognosis of recurrent HCC post-transplant, even in the current era, and that these patients mostly die from cancer, the potential for immunosuppression to inhibit the response to immunotherapy will also need to be considered. Last, to the best of our knowledge, this study is the first to independently associate the use of mTORi in the post-recurrence regimen with lower rates of mortality after accounting for antitumour treatment modality and other major prognostic determinants. The hypothesis that mTORi may have antitumour properties is not novel. It is important to note that the SILVER trial, a randomized controlled study evaluating the addition of sirolimus, an mTOR inhibitor, to the immunosuppressive regimen of liver transplant recipients with HCC, did not demonstrate a significant benefit.[16] However, the main endpoint of this study was the recurrence of HCC after LT and not the survival of patients with recurrence. Other small retrospective studies have also suggested that the addition of everolimus to sorafenib may improve survival of patients with recurrent HCC.[17,18] Our study provides an additional basis for setting up a randomized controlled trial, again stressing the need for international collaboration. Academic scientific societies will have a key role to play in coordinating and promoting such efforts.

There are several limitations to our study. First, due to insufficient data on tumour volume and number of nodules for each organ involved, we cannot exclude an allocation bias favouring curative approaches, as those were probably applied to patients with limited tumour burden. The benefit of radical approaches for patients with two or more organs involved, and for patients with bone, peritoneal or other extrahepatic involvement could not be formally assessed because of small sample sizes. However, the observation of increased OS with surgery in selected patients suggests that management of post-LT recurrent HCC should differ from the current paradigm of "systemic-only" treatment in non-transplanted patients with extrahepatic HCC. Second, the determination of patient-related risk factors for late recurrences was limited by inconsistently reported data on alcohol relapse and recurring fibrosis, highlighting the need for more systematic and standardized assessment after LT, and the lack of a comparative cohort of LT patients without recurrence. Third, our study includes patients mostly treated before the advent of immunotherapy-based combinations for HCC. Fourth, the retrospective nature of our study does not provide a sufficient level of evidence to recommend incorporating mTORi into the immunosuppressive regimen of patients with HCC recurrence. However, it strongly supports a randomized controlled trial, while acknowledging the challenges of standardizing immunosuppressive regimens in this setting. Fifth, the choice of RECIST-like cut-offs for categorization of AFP kinetics was arbitrary and these cut-offs are neither validated nor clinically used for AFP, which could limit the generalizability of our findings. However, an additional sensitivity analysis using 50% increases and decreases as cut-offs consistently showed similar results, demonstrating significant associations between AFP dynamics and time to recurrence. Sixth, a major limitation of our study is the use of explant pathology rather than pre-transplant imaging to assess adherence to Milan and AFP criteria. This bias may limit the generalizability of our findings regarding the impact of adherence to selection models. Finally, we were unable to analyse the impact of trough levels of immunosuppressants and therefore are not able to propose specific recommendations in this regard.

In conclusion, recurrent HCC after LT is a different entity from HCC in non-transplanted patients, with significant

heterogeneity in phenotypes of the disease in terms of time to recurrence, anatomical distribution, and therapeutic paradigms. The extension of selection criteria for transplantation did not impact post-recurrence outcomes but there is a signal of increased earlier recurrences that calls for caution. Surgical/ablative approaches with curative intent should be discussed for recurrence confined to only one organ, including the liver, the lungs, or lymph nodes. The development of new approved therapies for HCC after 2016 did not improve outcomes in this special population. mTORi may have beneficial effects beyond their role in CNI management. The management of HCC after LT is a major unmet need that should be addressed in dedicated clinical trials.

Affiliations

[1]*CHU Lille, Maladies de l'Appareil Digestif, Hôpital Huriez, Lille, France;* [2]*Université de Lille, Inserm, U1286 - INFINITE - Institute for Translational Research in Inflammation, Lille, France;* [3]*AP-HP, Hôpital Paul Brousse, Centre Hépato-Biliaire, Inserm UMR-S 1193, Université Paris-Saclay, Villejuif, France;* [4]*CHU Montpellier, Oncologie Médicale, Hôpital Saint-Eloi, Montpellier, France;* [5]*CHU Montpellier, Chirurgie hépatique, biliaire, pancréatique et transplantation, Hôpital Saint-Eloi, Montpellier, France;* [6]*CHU Montpellier, Hépato-gastro-entérologie, Unité d'Hépatologie et de Transplantation Hépatique, Hôpital Saint-Eloi, Institut de Génétique Moléculaire de Montpellier, Université de Montpellier, CNRS, Montpellier, France;* [7]*CHU Lille, Chirurgie Digestive et Transplantations, Hôpital Huriez, Lille, France;* [8]*CHU Rennes, Chirurgie hépatobiliaire et digestive, Hôpital Pontchaillou, Rennes, France;* [9]*CHU Rennes, Service des Maladies du Foie, Hôpital Pontchaillou, Rennes, France;* [10]*Service de chirurgie générale, hépatique, endocrinienne et transplantation – Hôpitaux universitaires de Strasbourg, France;* [11]*Service de chirurgie générale, hépatique, endocrinienne et transplantation – Hôpitaux universitaires de Strasbourg, Laboratoire ICube, INSERM UMR7357, Université de Strasbourg, Strasbourg, France;* [12]*AP-HP Sorbonne Université, Hôpital Universitaire Pitié-Salpêtrière, Service d'Hépato-gastroentérologie, INSERM UMR 1138, Centre de recherche des Cordeliers, Paris, 75006, France;* [13]*Service d'Hépato-gastro-entérologie, Unité Médicale de Transplantation Hépatique, Hôpital Pitié Salpêtrière, AP-HP, Paris, France;* [14]*AP-HP Sorbonne Université, Hôpital Universitaire Pitié-Salpêtrière, Service de Chirurgie Hépato-biliaire et Transplantation Hépatique, Paris, France;* [15]*Université Paris-Est Créteil, UPEC, INSERM, U955, Team "Virus Hépatologie Cancer", Service d'Hépatologie, Hôpital Henri Mondor, AP-HP, Créteil, France;* [16]*Service de Chirurgie Digestive et Hépato-Bilio-Pancréatique, Hôpital Henri Mondor, AP-HP, Université Paris-Est Créteil, UPEC, INSERM, U955, Team "Virus Hépatologie Cancer", Créteil, France;* [17]*CHU de Bordeaux, Hôpital Haut-Lévêque, Service de Chirurgie hépatobiliaire et de transplantation hépatique, Bordeaux, France;* [18]*CHU de Bordeaux, Hôpital Haut-Lévêque, Service de Chirurgie hépatobiliaire et de transplantation hépatique, Inserm UMR 1312, Bordeaux Institute of Oncology (BRIC), Université de Bordeaux, Bordeaux, France;* [19]*Service d'hépatologie et transplantation hépatique, Hôpital Beaujon, APHP, Clichy, France;* [20]*Service de Chirurgie hépato-bilio-pancréatique et transplantation hépatique, Hôpital Beaujon, APHP, Clichy, France;* [21]*Service d'Hépatologie, Lyon Liver Institute, IHU Everest, Hospices Civils de Lyon, Lyon, France;* [22]*Service d'hépatologie, CHU Rangueil, Toulouse, France et Université de Toulouse, France;* [23]*Département de néphrologie et transplantation d'organes, Hôpital Rangueil, CHU de Toulouse, Axe TImE, INSERM UMR 1291, Toulouse Institute for Infectious and Inflammatory Diseases (Infinity), Université Paul Sabatier, Toulouse, France;* [24]*Univ. Grenoble Alpes, Service d'hépato-gastroentérologie et d'oncologie digestive, CHU Grenoble Alpes, Grenoble, France;* [25]*CHU de Tours, Hôpital Trousseau, Service d'hépato-gastroentérologie, Université de Tours, Tours, France;* [26]*CHU de Tours, Hôpital Trousseau, Service de Chirurgie Digestive, Oncologique et Endocrinienne, Transplantation Hépatique, Tours, France;* [27]*IHU Méditerranée Infection, MEPHI, Aix-Marseille Université, Unité hépatologie, Hôpital de la Timone, Assistance Publique-Hôpitaux de Marseille (APHM), Marseille, France;* [28]*Service de Chirurgie et Transplantation Hépatique. Assistance Publique Hôpitaux de Marseille, Aix Marseille Université, Marseille France, France;* [29]*Fédération des spécialités digestives, Hôpital Edouard Herriot, Hospices civils de Lyon, et Université Claude Bernard Lyon 1, Lyon, France*

Abbreviations

AFP, alpha-foetoprotein; BSC, best supportive care; CNI, calcineurin inhibitor; EBRT, external beam radiation therapy; HCC, hepatocellular carcinoma; LRT, locoregional therapy; LT, liver transplantation; MELD, model for end-stage liver disease; MMF, mycophenolate mofetil; mTORi, mTOR inhibitor; OS, overall survival; TACE, transarterial chemoembolization; TKI, tyrosine kinase inhibitor.

Financial support

The authors did not receive any funding for this research from agencies in the public, private, or non-profit sectors.

Conflicts of interest

MN received travel and congress fees, from Roche, Astra-Zeneca, AbbVie and Gilead, consulting fees from Roche, Astra-Zeneca, and speaker honoraria from Roche, Astra-Zeneca, and Astellas. PH-D received travel and congress fees, and speaker honoraria from Gilead, Chiesi, Sandoz and Biotest. MA received consulting fees from AstraZeneca and Roche, and speaker honoraria from AstraZeneca, Bayer, AbbVie, Gilead, Roche, and Sirtex. NK has received speaker's fees and participated to advisory boards for Alexion, Astellas, Astra-Zeneca, Biotest, BMS, CSL Behring, Chiesi, Eledon, ExeViR, Gilead, Grifols, Hansa, MSD, GlaxoSmithKline, Pierre Fabre, Medison, Neovii, New Bridge, Roche, Sanofi, Sandoz, Synklino, Takeda, Zydus. L.E. received travel and congress fees, and honoraria for lectures, and presentations from Gilead, Ipsen, Echosens and Abbvie.

Please refer to the accompanying ICMJE disclosure forms for further details.

Authors' contributions

GL, MN and SD conceived and designed the study. MN, ML and CH collected the data. All authors provided the data. MN, ML, CH and GL performed the analysis. MN, GL and SD drafted the work. All authors contributed to the interpretation of the findings. All authors critically revised the study for intellectual content and approved the final version for publication. All authors agree to be accountable for all aspects of the work by ensuring that questions related to the accuracy or integrity of any part of this study are appropriately investigated and resolved.

Data availability

Data are available on request.

Declaration of generative AI and AI-assisted technologies in the manuscript preparation process

During the preparation of this work the authors used OpenAI's ChatGPT (free version) in order to improve the fluency of the English language, conciseness of the abstract. After using this tool, the authors carefully reviewed and edited the content as needed and take full responsibility for the content of the final text.

Supplementary data

Supplementary data to this article can be found online at https://doi.org/10.1016/j.jhepr.2026.102062.

References

Author names in bold designate shared co-first authorship

[1] Adam R, Karam V, Cailliez V, et al. 2018 Annual Report of the European liver transplant registry (ELTR) - 50-year evolution of liver transplantation. Transpl Int 2018;31:1293–1317. https://doi.org/10.1111/tri.13358.

[2] Reig M, Forner A, Rimola J, et al. BCLC strategy for prognosis prediction and treatment recommendation: the 2022 update. J Hepatol 2022;76:681–693. https://doi.org/10.1016/j.jhep.2021.11.018.

[3] European Association for the Study of the Liver. EASL Clinical Practice Guidelines on the management of hepatocellular carcinoma. J Hepatol 2025;82:315–374. https://doi.org/10.1016/j.jhep.2024.08.028.

[4] Duvoux C, Roudot-Thoraval F, Decaens T, et al. Liver transplantation for hepatocellular carcinoma: a model including alpha-fetoprotein improves the performance of Milan criteria. Gastroenterology 2012;143:986–994. https://doi.org/10.1053/j.gastro.2012.05.052. e3; quiz e14-5.

[5] Brusset B, Dumortier J, Cherqui D, et al. Liver transplantation for hepatocellular carcinoma: a Real-Life comparison of Milan criteria and AFP model. Cancers 2021;13. https://doi.org/10.3390/cancers13102480.
[6] Kodali S, Kulik L, D'Allessio A, et al. The 2024 ILTS-ILCA consensus recommendations for liver transplantation for hepatocellular carcinoma and intrahepatic cholangiocarcinoma. Liver Transplant n.d.:10.1097/LVT.0000000000000589. https://doi.org/10.1097/LVT.0000000000000589.
[7] Mehta N, Heimbach J, Harnois DM, et al. Validation of a risk estimation of tumor recurrence after transplant (RETREAT) score for hepatocellular carcinoma recurrence after liver transplant. JAMA Oncol 2017;3:493–500. https://doi.org/10.1001/jamaoncol.2016.5116.
[8] Halazun KJ, Najjar M, Abdelmessih RM, et al. Recurrence after liver transplantation for hepatocellular carcinoma: a new MORAL to the story. Ann Surg 2017;265:557–564. https://doi.org/10.1097/SLA.0000000000001966.
[9] **Costentin C**, **Pinero F**, Degroote H, et al. R3-AFP score is a new composite tool to refine prediction of hepatocellular carcinoma recurrence after liver transplantation. JHEP Rep 2022;4:100445. https://doi.org/10.1016/j.jhepr.2022.100445.
[10] Li Z, Chen IC-Y, Centonze L, et al. Analysis of treatment benefits and prognostic factors for posttransplant HCC recurrence in a large Euro-American-Asian cohort. Liver Transplant 2025;31:450. https://doi.org/10.1097/LVT.0000000000000501.
[11] Belli LS, Perricone G, Adam R, et al. Impact of DAAs on liver transplantation: major effects on the evolution of indications and results. An ELITA study based on the ELTR registry. J Hepatol 2018;69:810–817. https://doi.org/10.1016/j.jhep.2018.06.010.
[12] **Belli LS**, **Duvoux C**, Berenguer M, et al. ELITA consensus statements on the use of DAAs in liver transplant candidates and recipients. J Hepatol 2017;67:585–602. https://doi.org/10.1016/j.jhep.2017.03.006.
[13] Magyar CTJ, Perera S, Rajendran L, et al. Comparative outcome analysis of lenvatinib *Vs.* Sorafenib for recurrence of hepatocellular carcinoma after liver transplantation. Transplantation 2024. https://doi.org/10.1097/TP.0000000000005240.
[14] Kudo M, Finn RS, Qin S, et al. Lenvatinib *vs.* sorafenib in first-line treatment of patients with unresectable hepatocellular carcinoma: a randomised phase 3 non-inferiority trial. Lancet 2018;391:1163–1173. https://doi.org/10.1016/S0140-6736(18)30207-1.
[15] Kahramangil D, Zarrinpar A, Sahin I. Immune checkpoint inhibitors in the post-transplant landscape of hepatocellular carcinoma: a systematic literature review. Liver Transpl 2024. https://doi.org/10.1097/LVT.0000000000000550.
[16] Geissler EK, Schnitzbauer AA, Zulke C, et al. Sirolimus Use in liver transplant recipients with hepatocellular carcinoma: a randomized, multicenter, Open-Label phase 3 trial. Transplantation 2016;100:116–125. https://doi.org/10.1097/TP.0000000000000965.
[17] Invernizzi F, Iavarone M, Zavaglia C, et al. Experience with early sorafenib treatment with mTOR inhibitors in hepatocellular carcinoma recurring after liver transplantation. Transplantation 2020;104:568–574. https://doi.org/10.1097/TP.0000000000002955.
[18] Nitta H, Younes A, El-Domiaty N, et al. High trough levels of everolimus combined to sorafenib improve patients survival after hepatocellular carcinoma recurrence in liver transplant recipients. Transpl Int 2021;34:1293–1305. https://doi.org/10.1111/tri.13897.

Keywords: Liver transplantation; Hepatocellular carcinoma; Recurrence; Extended criteria; Immunosuppressive regimen.

Received 13 March 2026; received in revised form 25 September 2026; accepted 2 October 2026; Available online 9 October 2026

Journal of Hepatology, Volume *8*

Supplemental information

The landscape of hepatocellular carcinoma recurrence after liver transplantation: A nationwide 14-year study

Massih Ningarhari, Mathurine Leray, Audrey Coilly, Eric Vibert, Eric Assenat, Astrid Herrero, Jose Ursic-Bedoya, Camille Henry, Line-Carolle Ntandja-Wandji, Elise Lemaitre, Alexandre Louvet, Philippe Mathurin, Emmanuel Boleslawski, Fabien Robin, Thomas Uguen, Pauline Houssel-Debry, Camille Besch, François Faitot, Manon Allaire, Filomena Conti, Olivier Scatton, Giuliana Amaddeo, Daniele Sommacale, Jean-Baptiste Hiriart, Louis Marjary, Faiza Chermak, Laurence Chiche, Olivier Roux, Claire Francoz, Federica Dondero, Teresa Antonini, Domitille Erard, Jean-Marie Peron, Nassim Kamar, Bleuenn Brusset, Thomas Decaens, Laure Elkrief, Hélène Barraud, Ephrem Salamé, René Gerolami, Sophie Chopinet, Jérôme Dumortier, Olivier Boillot, Sébastien Dharancy, Guillaume Lassailly, and on behalf of the GReF2 study group

The landscape of hepatocellular carcinoma recurrence after liver transplantation: A nationwide 14-year study

Massih Ningarhari, Mathurine Leray, Audrey Coilly, Eric Vibert, Eric Assenat, Astrid Herrero, Jose Ursic-Bedoya, Camille Henry, Line-Carolle Ntandja-Wandji, Elise Lemaitre, Alexandre Louvet, Philippe Mathurin, Emmanuel Boleslawski, Fabien Robin, Thomas Uguen, Pauline Houssel-Debry, Camille Besch, François Faitot, Manon Allaire, Filomena Conti, Olivier Scatton, Giuliana Amaddeo, Daniele Sommacale, Jean-Baptiste Hiriart, Louis Marjary, Faiza Chermak, Laurence Chiche, Olivier Roux, Claire Francoz, Federica Dondero, Teresa Antonini, Domitille Erard, Jean-Marie Peron, Nassim Kamar, Bleuenn Brusset, Thomas Decaens, Laure Elkrief, Hélène Barraud, Ephrem Salamé, René Gerolami, Sophie Chopinet, Jérôme Dumortier, Olivier Boillot, Sébastien Dharancy, Guillaume Lassailly, on behalf of the GReF2 study group

Table of contents

Fig. S1 3

Fig. S2 4

Fig. S3. 5

Fig. S4 6

Fig. S5. 7

Table S1 8

Table S2 9

Table S3 10

Table S4 11

Table S5 12

Table S6 13

Table S7 14

Table S8. 15

Table S9 16

Table S10 17

Table S11 18

Table S12 19

Table S13 20

Fig. S1 – Study Flow-chart

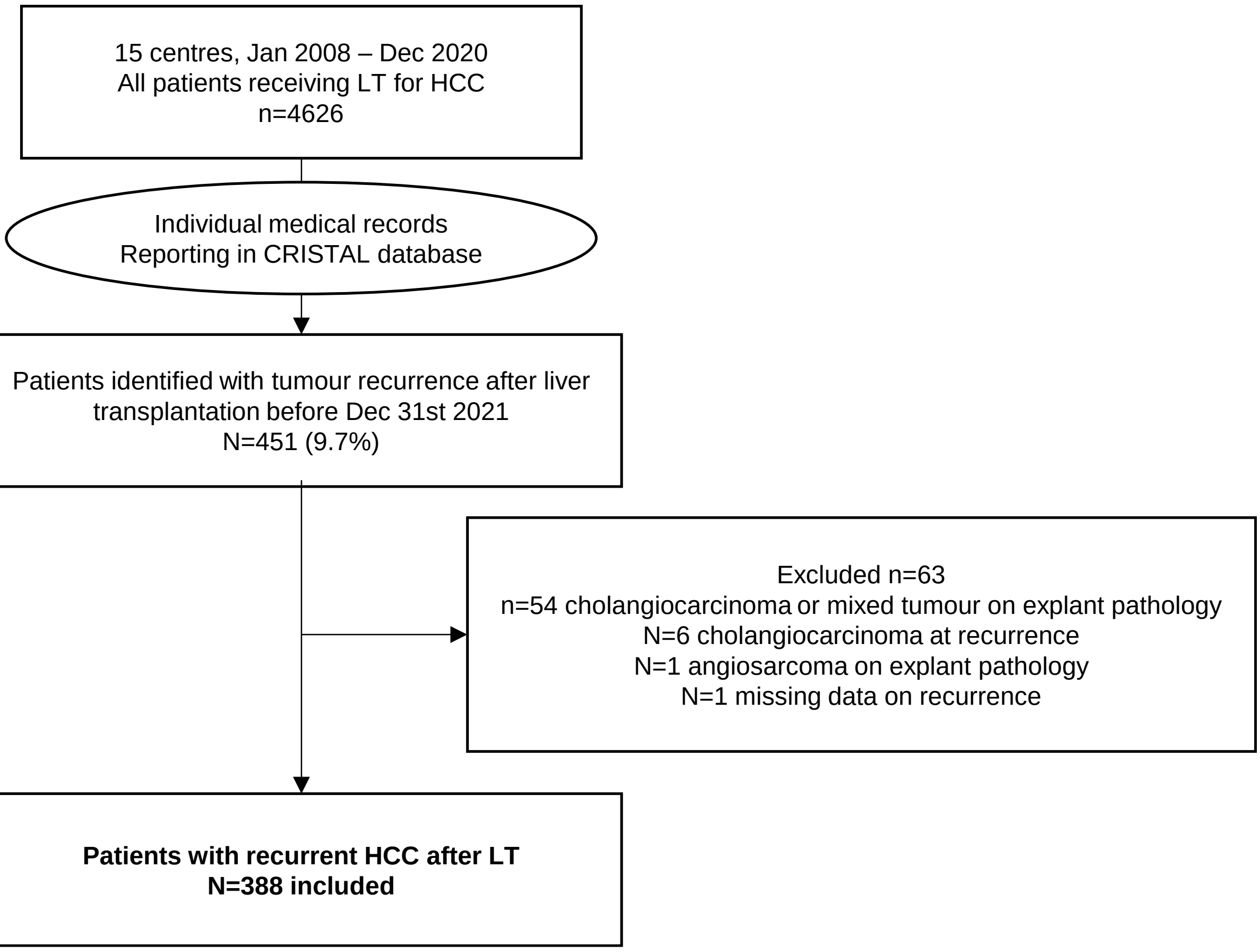

LT, liver transplantation. HCC, hepatocellular carcinoma**.**

Fig. S2 – Covariate balance after propensity score matching, to compare patients treated with curative versus non-curative intent

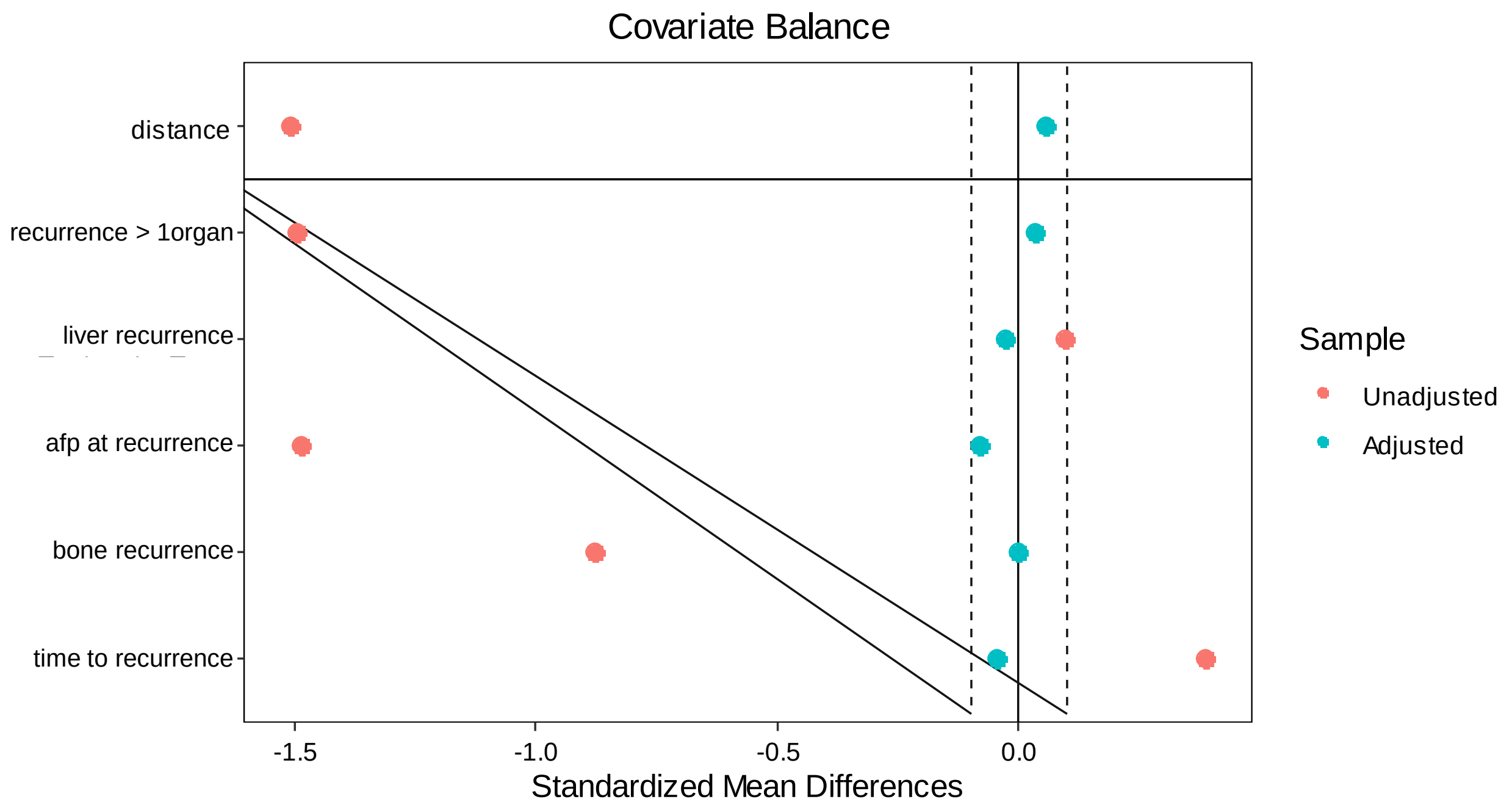

Fig. S3 – Benefit of treatments with curative intent in patients with only one organ affected by recurrence.

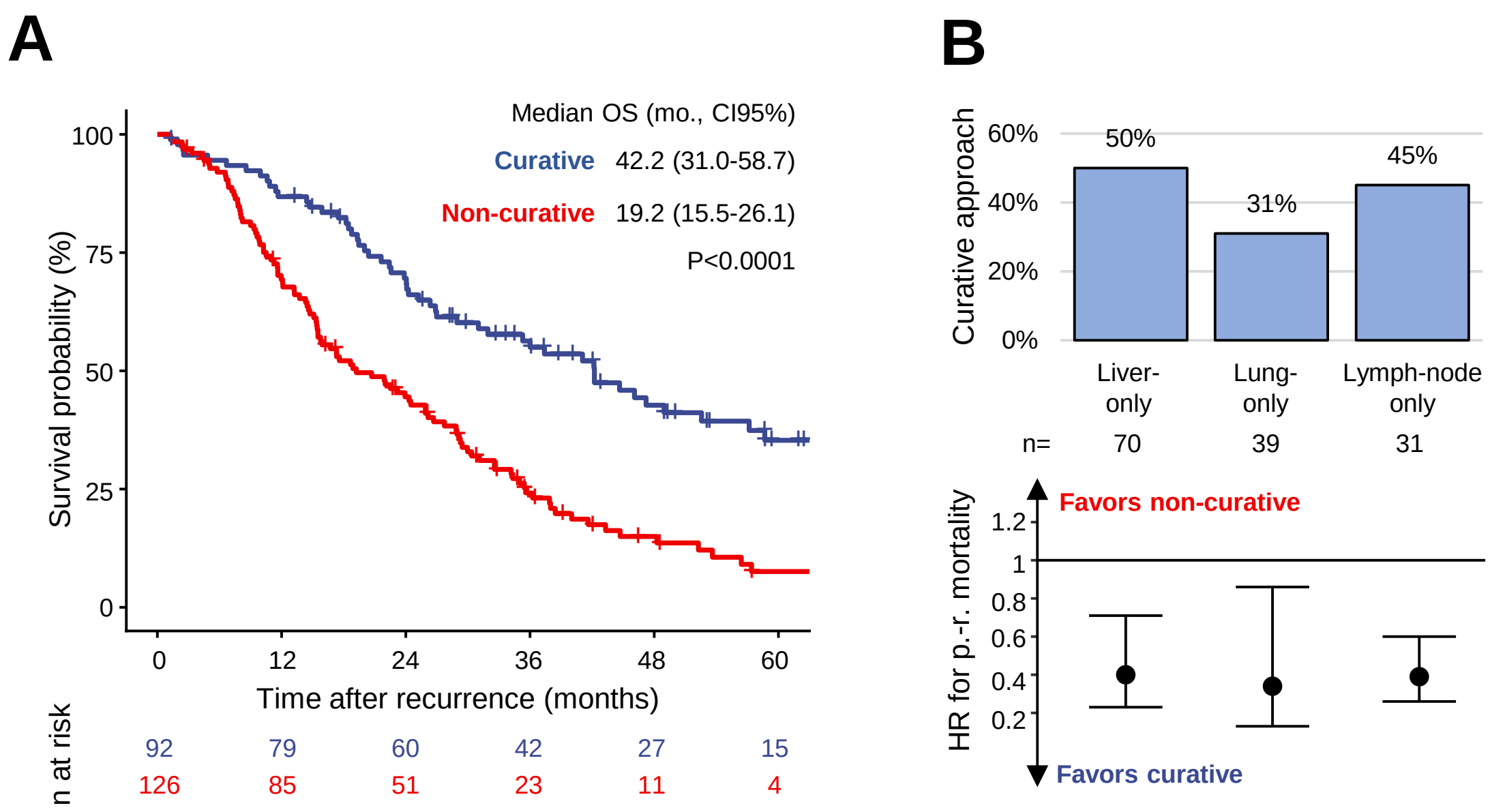

(A) Post-recurrence overall survival of patients according to the first treatment applied, compared with the log-rank test. (B) Proportion of patients (top) receiving surgical and ablative treatments, and benefit (bottom) of such treatment compared to systemic treatment first, according to the anatomical site of recurrence, using Cox regression model stratified by organ (dots represent the hazard ratio and bars the 95% confidence interval). Level of significance set at $p \leq 0.05$. n, number of patients with available data. OS, overall survival. Mo., months. CI95%, 95% confidence interval. NR, not reached. HR, hazard ratio. p-r., post-recurrence.

Fig. S4 – Distribution of scores according to the AFP model before and after the adoption as selection criteria in 2013

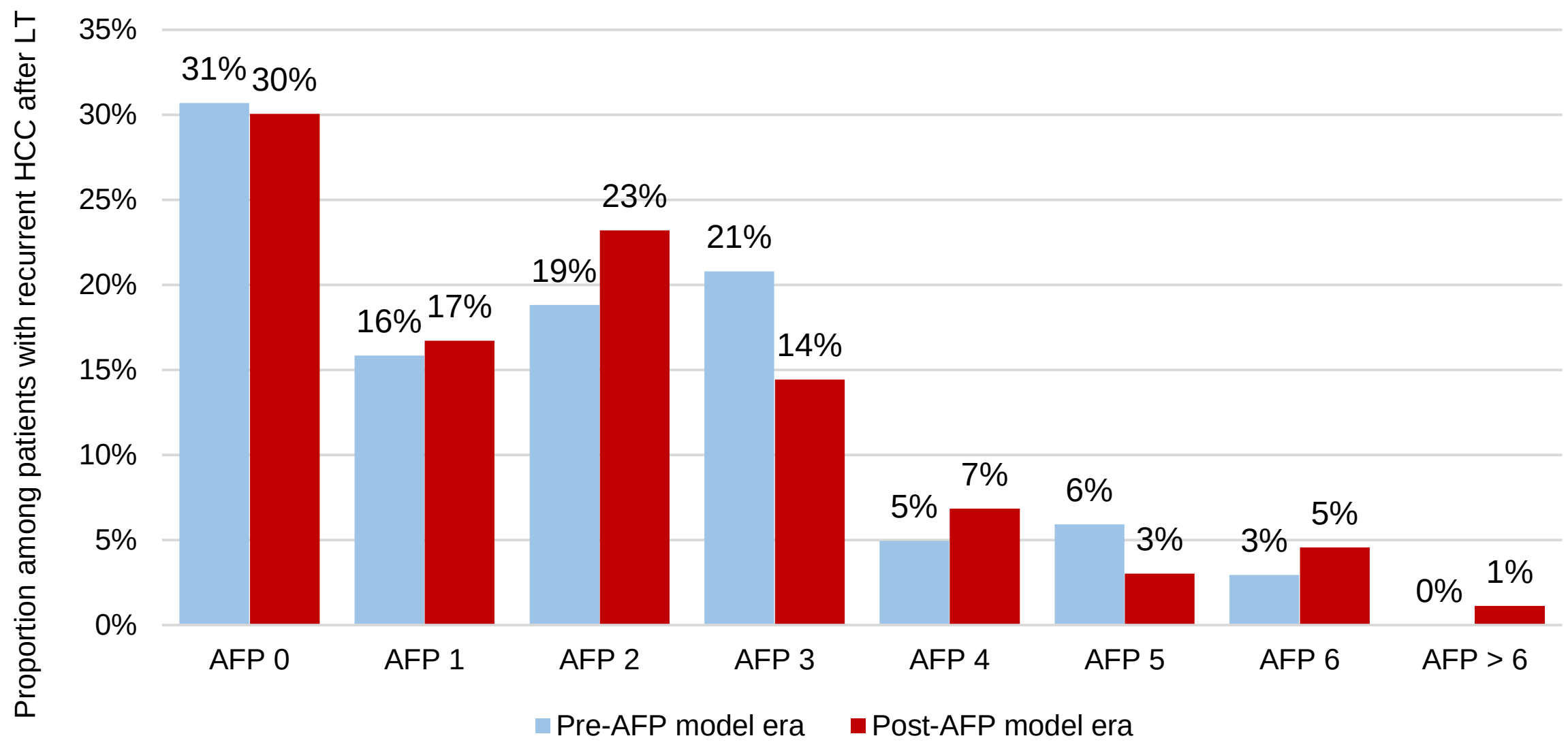

Fig. S5 – Association of pretransplant tumour characteristics with post-recurrence survival.

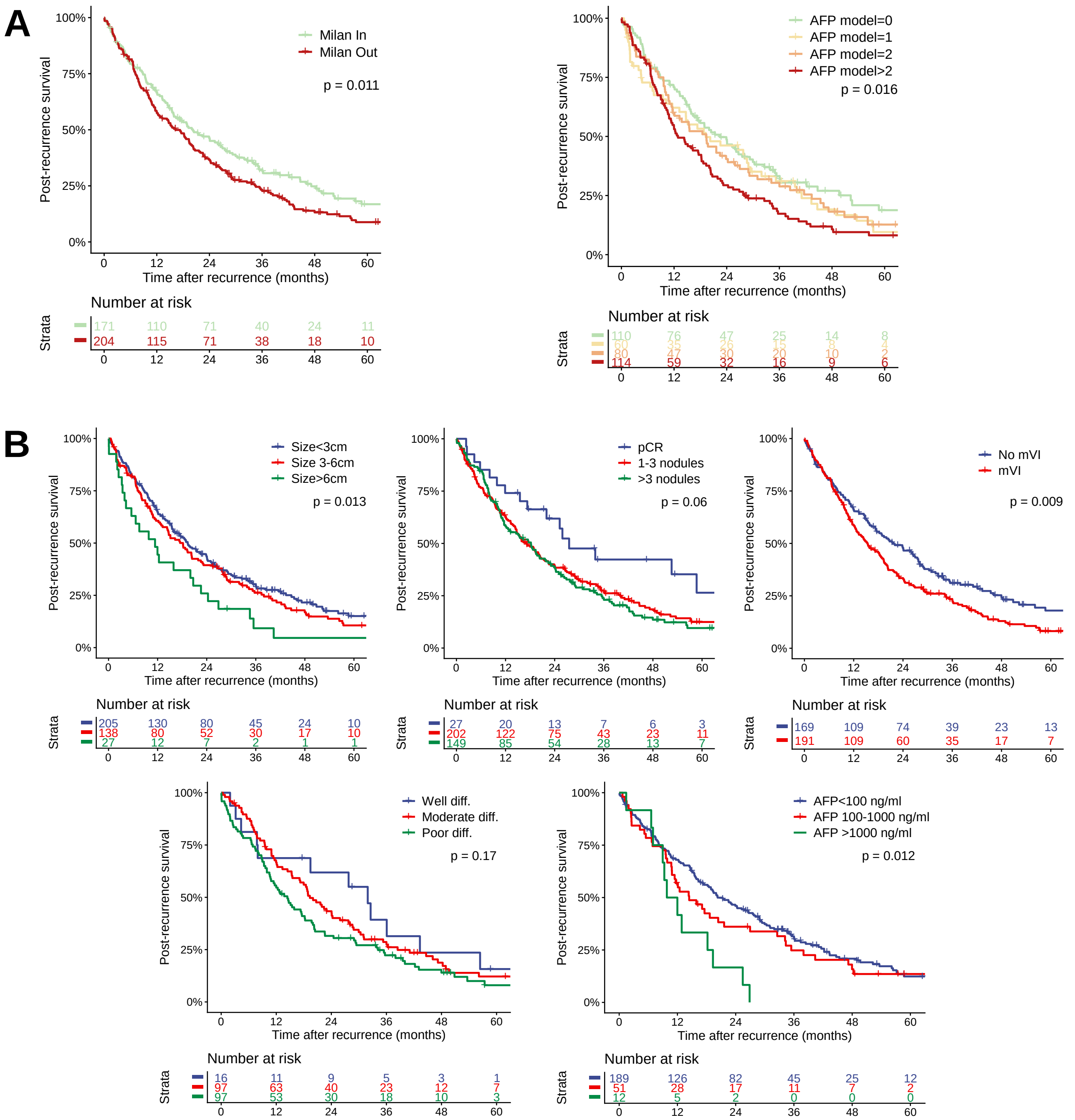

Post-recurrence overall survival according to (A) Milan criteria and AFP model score, and (B) individual components of the AFP model, vascular invasion and tumour differentiation, comparisons with the log-rank test. Level of significance set at p≤0.05. pCR, pathological complete response; mVI, microvascular invasion; diff., tumour differentiation

Table S1 – The AFP model criteria (adapted from Duvoux et al., Gastroenterology 2012)

Variables	β coefficient	Hazard ratio	Points
Largest diameter			
⩽3 cm	0	1	0
3–6 cm	0.272	1.31	1
>6 cm	1.347	3.84	4
Number of nodules			
1–3	0	1	0
4 and more	0.696	2.01	2
AFP level (ng/ml)			
⩽100	0	1	0
[100–1000]	0.668	1.95	2
>1000	0.945	2.57	3

The score is calculated by adding the individual points for each obtained variable. A cut-off of 2 separates between patients at high and low risk of recurrence.

Table S2 – Pre-transplant characteristics of patients with recurrent hepatocellular carcinoma after liver transplantation

	N	
**Male sex**	388	332 (85.6)
**Underlying liver disease and risk factors**		
Cirrhosis	388	367 (94.6)
Alcohol-related	388	209 (54.3)
BMI (kg/m²)	263	26.5 (23.3-30)
Diabetes	380	112 (29.5)
Hepatitis C	388	148 (38.1)
Hepatitis B	388	40 (10.3)
Other	388	21 (5.4)
**Time between HCC diagnosis to LT**	287	19.8 (12.5-33.1)
**Bridging therapy**		
Any	388	312 (80.4)
TACE	312	271 (86.9)
Other LRT (PA or EBRT)	312	69 (22.1)
Systemic	312	21 (6.7)
**Serum αFP dynamics on waitlist**		
αFP at listing (ng/ml)	359	8.9 (4.2-32)
Maximal αFP (ng/ml)	355	18 (6-85.5)
Last αFP pre-LT (ng/ml)	379	16 (5-100.5)
Stable αFP	355	171 (48.2)
Decreasing αFP >30%	355	66 (18.6)
Increasing αFP >20%	355	118 (33.2)
**Age at LT**	388	60.4 (55.2-63.8)
**LT in the AFP model era**	388	271 (70)

N, patients with available data. Quantitative variables are presented as median (interquartile range, IQR), qualitative variables as n (%). Percentage calculated within each line on patients with available data. BMI, body mass index. TACE, transarterial chemoembolization. LRT, locoregional therapy. PA, percutaneous ablation. EBRT, external-beam radiotherapy. αFP, alpha-foetoprotein. LT, liver transplantation.

Table S3 – Explant characteristics of patients with recurrent hepatocellular carcinoma after liver transplantation

	**N**	
**Number of nodules**	378	
0 (pathological complete response)		27 (7.1)
1-3		202 (53.4)
4+		149 (39.4)
**Size of the largest nodule**	370	
<3cm		205 (55.4)
3-6cm		138 (37.3)
>6cm		27 (7.3)
**Microvascular invasion**	360	191 (53.1)
**Nuclear grade 3**	210	97 (46.2)
**Milan-In**	375	171 (45.6)
**AFP model**	364	
0		110 (30.2)
1		60 (16.5)
2		80 (22.0)
>2		114 (31.3)

N, patients with available data. Qualitative variables are presented as n (%). Percentage calculated within each line on patients with available data.

Table S4 – Tumour burden at recurrence according to first treatment applied (all patients)

		Curative first (resection/ablation)	Locoregional first (incl. combination)	Systemic first	Best supportive care
	**N=**	**99**	**55**	**188**	**45**
**Organs**					
Liver	152	39 (25.7)	23 (15.1)	73 (48.0)	17 (11.2)
Lungs	123	15 (12.2)	6 (4.9)	82 (66.7)	20 (16)
Bones	100	9 (9.0)	16 (16.0)	55 (55.0)	20 (20.0)
Lymph nodes	92	15 (16.3)	8 (8.7)	58 (63.0)	11 (12.0)
Adrenal	29	11 (37.9)	2 (6.9)	14 (48.3)	2 (6.9)
Peritoneal/seeding	68	14 (20.6)	5 (7.4)	36 (52.9)	13 (19.1)
Others	55	16 (29.1)	10 (18.2)	23 (41.8)	6 (10.9)
**Distribution**					
Liver-only	77	35 (45.5)	14 (18.2)	22 (28.6)	6 (7.8)
Extrahepatic-only	235	60 (25.5)	32 (13.6)	115 (48.9)	28 (11.9)
Both	75	4 (5.3%)	9 (12.0)	51 (68.0)	11 (14.7)
**Number of organs**					
Single organ	238	92 (38.7)	42 (17.6)	84 (35.3)	20 (8.4)
Two organs	106	5 (4.7)	13 (12.3)	74 (69.8)	14 (13.2)
Three or more	42	2 (4.8)	0 (0)	29 (69.0)	11 (26.2)
**AFP at recurrence (ng/ml)**	340	8 (3-47)	36 (4-339)	14 (4-180)	29 (6-392)

N, patients with available data. Quantitative variables are presented as median (interquartile range, IQR), qualitative variables as n (%). BSC, best supportive care. Percentage calculated within each line on patients with available data.

Table S5 – Tumour burden at recurrence according to first treatment applied (patients with only one organ affected by recurrence, excluding those only eligible to best supportive care)

		Curative first (resection/ablation)	Non-curative first (excl. BSC)	
	**N**	**92**	**126**	**P**
**Organs**				
Liver	70	35 (50.0)	35 (50.0)	0.14
Lungs	39	12 (30.8)	27 (69.2)	0.16
Bones	36	5 (13.9)	31 (86.1)	0.0003
Lymph nodes	31	14 (45.2)	17 (54.8)	0.87
Adrenal	14	11 (78.6)	3 (21.4)	0.004
Peritoneal/seeding	15	10 (66.7)	5 (33.3)	0.09
Others	27	16 (59.3)	11 (40.7)	0.34
Extrahepatic-only	148	57 (38.5)	91 (61.5)	
**AFP at recurrence (ng/ml)**	196	8 (3-32)	11 (4-183)	0.13

N, patients with available data. Quantitative variables are presented as median (interquartile range, IQR), qualitative variables as n (%). BSC, best supportive care. Percentage calculated within each line on patients with available data. Comparisons and p values using $\chi 2$ test for qualitative variables, and Wilcoxon rank-sum (i.e. Mann-Whitney U-test) test for quantitative variables, level of significance set at $p \leq 0.05$.

Table S6 – Systemic therapies used for recurrent hepatocellular carcinoma after liver transplantation

	**L1**	**L2**	**L3**	**L4**	**L5**
**N=**	283	150	70	34	11
Sorafenib	232 (82.0)	14 (9.3)	1 (1.4)	0	2 (18.2)
Lenvatinib	28 (9.9)	9 (6.0)	5 (7.1)	10 (29.4)	5 (45.5)
Chemotherapy	14 (4.9)	36 (24.0)	25 (35.7)	6 (17.6)	2 (18.2)
Regorafenib	1 (0.4)	60 (40.0)	14 (20)	2 (5.9)	0
Cabozantinib	1 (0.4)	25 (16.7)	19 (27.1)	7 (20.6)	1 (9.1)
Immunotherapy	3 (1.1)	3 (2)	2 (2.9)	7 (20.6)	1 (9.1)
Others	4 (1.3)	3 (2)	4 (5.7)	2 (5.9)	0

N, patients with available data. Qualitative variables are presented as as n (%). Percentage calculated within each column on patients with available data. L1 to L5, First to fifth line of systemic treatment.

Table S7 - Characteristics of patients according to the year of recurrence of hepatocellular carcinoma after liver transplantation

**Year of recurrence**	**2008-2015**	**2016-2017**	**2018-**	
**N**	**133**	**109**	**146**	p
**Time to recurrence**				0.0007
<6 months	24 (18.0)	11 (10.1)	13 (8.9)	
6-36 months	82 (61.7)	70 (64.2)	86 (58.9)	
36-60 months	23 (17.3)	19 (17.4)	20 (13.7)	
>60 months	4 (3.0)	9 (8.3)	27 (18.4)	
**1st treatment for recurrence**				0.27
Curative intent	32 (24.0)	24 (22.0)	44 (30.1)	
Non-curative intent	101 (76.0)	85 (78.0)	102 (69.9)	
**Any systemic 1st-line**	**99 (74.4)**	**83 (76.1)**	**101 (69.2)**	0.35
Sorafenib	82 (82.8)	79 (95.2)	71 (70.3)	
Lenvatinib	2 (2.0)	2 (2.4)	24 (23.8)	
Other TKI	0	0	2 (2.0)	
IO	0	0	3 (3.0)	
Chemotherapy	13 (13.1)	2 (2.4)	1 (1.0)	
Other drugs	2 (2.0)	0	0	
**Access to 2nd line**	40 (40.4)	49 (59.0)	61 (60.4)	0.11
Sorafenib	9 (22.5)	0	5 (8.2)	
Lenvatinib	0	1 (2.0)	8 (13.1)	
Regorafenib	6 (15.0)	29 (59.2)	25 (41.0)	
Cabozantinib	1 (2.5)	6 (12.2)	18 (29.5)	
IO	0	0	3 (4.9)	
Chemotherapy	22 (55.0)	12 (24.5)	2 (3.3)	
Other drugs	2 (5.0)	1 (2.0)	0	
**Access to 3rd line**	17 (17.2)	26 (31.3)	27 (26.7)	0.27
**Access to >3 lines**	10 (10.1)	10 (12.0)	13 (12.9)	0.81

N, number of patients with available data. Qualitative variables are presented as n (%). Percentage are calculated within each column on patients with available data. HCC, hepatocellular carcinoma**.** TKI, tyrosine kinase inhibitor. IO, immunotherapy (single or combination). Comparisons and p values using $\chi 2$ test for qualitative variables, level of significance set at $p \leq 0.05$.

Table S8 – Main characteristics of patients diagnosed after 2018 and treated with either sorafenib or lenvatinib as first-line systemic therapy.

	N	Sorafenib 71	Lenvatinib 24	P
**Time to recurrence (months)**	95	23.8 (13.4-36.8)	29.5 (11.1-49.9)	0.68
**Delay of recurrence**	95			0.4
<6 months		3 (4.2)	2 (8.3)	
6-36 months		49 (69)	13 (54.2)	
36-60 months		10 (14.1)	3 (12.5)	
>60 months		9 (12.7)	6 (25)	
**Time from recurrence to systemic therapy initiation**	95	2 (0.9-3.8)	6.5 (2.1-17.5)	0.008
**Recurrent liver disease**	93	10 (14.5)	3 (12.5)	
**Recurrent tumour distribution**	95			
Liver		29 (40.8)	3 (12.5)	0.02
Lungs		17 (23.9)	7 (29.2)	0.84
Bones		21 (29.6)	8 (33.3)	0.96
>1 organ affected		38 (53.5)	5 (20.8)	0.009
Liver-only		12 (16.9)	2 (8.3)	
Extrahepatic-only		42 (59.2)	21 (87.5)	
Both		17 (23.9)	1 (4.2)	
**AFP at recurrence (ng/ml)**	90	14 (5-94)	7 (3-64)	0.15
**Previous treatment**	95			
Surgery/Ablation		7 (9.9)	9 (37.5)	0.005
Non-curative locoregional treatment		9 (12.7)	7 (29.2)	0.12

N, patients with available data. Quantitative variables are presented as median (interquartile range, IQR), qualitative variables as n (%). Percentage calculated within each column on patients with available data. Comparisons and p values using $\chi 2$ test for qualitative variables, and Wilcoxon rank-sum (i.e. Mann-Whitney U-test) test for quantitative variables, level of significance set at $p \leq 0.05$.

Table S9 – Sensitivity analysis on patients with patients receiving LT before 2016, restricted on a 5-year post-LT window

**Selection era for LT**	**Before AFP model**	**After AFP model**	
**N**	**90**	**154**	**p**
**Time to recurrence**			
<6 months	13 (14.4)	17 (11.0)	**0.02**
6-36 months	48 (53.3)	109 (70.8)	
36-60 months	29 (32.2)	28 (18.2)	

N, patients with available data. Qualitative variables are presented as n (%). Percentage on patients with available data within each era of transplantation. LT, liver transplantation. Comparisons and p values using $\chi 2$ test for qualitative variables, level of significance set at $p \leq 0.05$.

Table S10 – Patients characteristics according to mTOR inhibitor introduction before recurrence

		mTORi before recurrence	No mTORi before recurrence	
	N=	104	266	P
**Tumour dynamics before LT**				
Any bridging therapy	370	86 (82.7)	211 (79.3)	0.56
AFP dynamics				
AFP at listing	344	9 (4-38)	9 (4-32)	0.97
Maximal AFP	339	23 (7-168)	16 (5-69)	0.03
Last AFP pre-LT	362	14 (6-88)	10 (4-46)	0.02
Stable AFP		39 (41.1)	124 (50.8)	0.24
Decreasing AFP >30%		22 (23.2)	43 (17.6)	
Increasing AFP >20%		34 (35.8)	77 (31.6)	
**Explant pathology**				
Number of nodules	361			<0.0001
0 (pCR)		2 (2)	25 (9.7)	
1-3		39 (38.2)	153 (59.1)	
4+		61 (59.8)	81 (31.3)	
Size of the largest nodule	353			0.1
<3cm		48 (48.5)	150 (59.1)	
3-6cm		40 (40.4)	89 (35)	
>6cm		11 (11.1)	15 (5.9)	
Microvascular invasion	343	59 (60.2)	121 (49.4)	0.09
Nuclear grade 3	199	29 (40.3)	61 (48)	0.47
Milan-In using explant	358	26 (25.5)	140 (54.7)	<0.0001
AFP model using explant	348			<0.0001
0		15 (15.5)	92 (36.7)	
1		11 (11.3)	46 (18.3)	
2		28 (28.9)	49 (19.5)	
>2		43 (44.3)	64 (25.5)	
**LT characteristics**				
Age at LT	370	60 (53-64)	61 (56-64)	0.54
LT after AFP model implementation	370	80	183	0.16
**IS management before recurrence**				
Time from LT to mTORi (months)		2.6 (1.6-7)	-	-
Time from mTORi to recurrence (months)		14.9 (6-26.1)	-	-

N, patients with available data. Quantitative variables are presented as median (interquartile range, IQR), qualitative variables as n (%). Percentage on patients with available data within each column. Comparisons and p values using $\chi 2$ test for qualitative variables, and Wilcoxon rank-sum (i.e. Mann-Whitney U-test) test for quantitative variables, level of significance set at $p \leq 0.05$. LT, liver transplantation. mTORi, mTOR inhibitor. pCR, pathological complete response.

Table S11 – Combined mTOR and calcineurin inhibitors management

		CNI interrupted before recurrence	CNI interrupted upon recurrence	CNI maintained after recurrence
	**N=293**	**36**	**71**	**186**
mTORi before recurrence	**70**	30 (42.9)	9 (12.9)	31 (44.3)
mTORi upon recurrence	**126**	4 (3.2)	61 (48.4)	61 (48.4)
mTORi not introduced	**97**	2 (2.1)	1 (1)	94 (96.9)

N, patients with available data. Qualitative variables are presented as n (%). Percentage on patients with available data within each line. mTORi, mTOR inhibitor. CNI, calcineurin inhibitor.

Table S12 – Characteristics at recurrence according to the use of mTOR inhibitors in post-recurrence immunosuppressive regimen

		With mTOR inhibitor	Without mTOR inhibitor	
	**N=**	**257**	**113**	**P**
**Time to recurrence (months)**	370	18 (9.8-34.3)	20.5 (11.8-46)	0.05
**Delay of recurrence**	370			0.12
<6 months		31 (12.1)	14 (12.4)	
6-36 months		169 (65.8)	62 (54.9)	
36-60 months		36 (14)	20 (17.7)	
>60 months		21 (8.2)	17 (15)	
**Age at recurrence**	370	62 (56-66)	63 (59-67)	0.04
**Recurrent liver disease**	357	43 (17.6)	20 (17.9)	1
**Recurrent tumour distribution**	370			
Liver		94 (36.6)	51 (45.1)	0.15
Lungs		79 (30.7)	39 (34.5)	0.57
Bones		66 (25.7)	29 (25.7)	1
>1 organ affected		91 (35.4)	51 (45.1)	0.1
Liver-only		52 (20.2)	22 (19.5)	0.1
Extrahepatic-only		163 (63.4)	62 (54.9)	
Both		42 (16.3)	29 (25.7)	
**AFP at recurrence**	327	14 (4-130)	9 (3-208)	0.65
**Treatment with curative intent**	370	66 (25.7)	28 (24.8)	0.96

N, patients with available data. Quantitative variables are presented as median (interquartile range, IQR), qualitative variables as n (%). Percentage on patients with available data within each column. Comparisons and p values using $\chi 2$ test for qualitative variables, and Wilcoxon rank-sum (i.e. Mann-Whitney U-test) test for quantitative variables, level of significance set at $p \leq 0.05$. LT, liver transplantation.

Table S13 – Factors associated with mortality after recurrence of hepatocellular carcinoma after liver transplantation (Cox regression)

	N	% missing	Univariable HR (CI95%)	Univariable p	Multivariable HR (CI95%)	Multivariable p
Age	388	0%	1 (0.98-1.01)	0.9	-	-
LT after AFP Criteria implementation	388	0%	1.13 (0.89-1.44)	0.3	-	-
Date of recurrence	388	0%				
2008-2015			Ref.		-	-
2015-2017			1.1 (0.84-1.43)	0.5	-	-
2018-			0.8 (0.61-1.05)	0.1	-	-
**Time to recurrence**	**388**	**0%**				
**<6 months**			**Ref.**		**Ref.**	
**6-36 months**			**0.63 (0.45-0.89)**	**0.007**	**0.59 (0.41-0.84)**	**0.004**
**36-60 months**			**0.4 (0.26-0.62)**	**<0.001**	**0.44 (0.27-0.69)**	**<0.001**
**>60 months**			**0.36 (0.21-0.61)**	**<0.001**	**0.34 (0.19-0.60)**	**<0.001**
PS grade >0	347	11%	2.27 (1.79-2.89)	<0.001	-	-
Serum αFP (by log^{10})	340	12%	1.37 (1.23-1.54)	<0.001	-	-
Organs affected by recurrence						
**Liver**	**388**	**0%**	**1.38 (1.1-1.73)**	**0.006**	**1.56 (1.19-2.04)**	**0.001**
Lungs	388	0%	1.24 (0.98-1.57)	0.07	-	-
**Bones**	**388**	**0%**	**1.71 (1.33-2.19)**	**<0.001**	**1.50 (1.14-1.98)**	**0.004**
**More than one organ**	**388**	**0%**	**2.02 (1.6-2.54)**	**<0.001**	**1.36 (1.04-1.78)**	**0.03**
Distribution of recurrence	388	0%				
Liver-only			Ref.		-	-
Extrahepatic only			0.86 (0.65-1.15)	0.32	-	-
Both			1.46 (1.02-2.07)	0.04	-	-
**Treatment with curative intent**	**387**	**0%**	**0.33 (0.24-0.44)**	**<0.001**	**0.43 (0.31-0.6)**	**<0.001**
**mTOR inhibitor**	**370**	**5%**	**0.67 (0.52-0.86)**	**0.001**	**0.62 (0.48-0.79)**	**<0.001**
CNI	295	24%	1.25 (0.96-1.63)	0.1	-	-
MMF	293	24%	1.12 (0.86-1.45)	0.4	-	-

The most relevant variables with rates of missing data <10% and significant association (threshold set p<0.05) with mortality in univariate analysis were introduced into the multivariate Cox model, level of significance set at p≤0.05. N, patients with available data. HR, hazard ratio. CI95%, 95% confidence interval. PS, performance status. CNI, calcineurin inhibitor. MMF, mycophenolate mofetil.
